# Supplementary material for: Validation of tRNA-derived fragments as diagnostic biomarkers in suspected acute stroke; limitations in analysis and quantification methods
Source: Mol Ther Nucleic Acids. 2025 May 5;36(2):102553. doi: 10.1016/j.omtn.2025.102553 (PMC12143625; doi:10.1016/j.omtn.2025.102553)
Supplement: Document S1. Figures S1–S6 and Tables S1 and S2 [file mmc1.pdf]

OMTN, Volume *36*

Supplemental information

Validation of tRNA-derived fragments as diagnostic biomarkers in suspected acute stroke; limitations in analysis and quantification methods

Tamar Woudenberg, M. Leontien van der Bent, Daphne A.L. van den Homberg, T. Truc My Nguyen, Marieke J.H. Wermer, Ido R. van den Wijngaard, Paul H.A. Quax, A. Yaël Nossent, and Nyika D. Kruyt

Table S1: Fragments and their contribution to the total reads. Percentages of total reads attributed to the chosen dominant fragment(s), the next most abundant fragment (highlighting the contribution of other prominent tRFs from the same isodecoder) and fragments detected in five or fewer samples (illustrating the proportion of reads derived from sequences with limited occurrence across the patient cohort) are shown. Percentages reflect reads mapped to the same genomic locus/loci as the dominant fragment.

Isodecoder	***Chosen fragment(s):*** *% of Total Reads*	***Next most common fragment:*** *% of Total Reads*	***Rare fragments** (<5 samples): % of Total Reads*
ArgTCG	51%	5%	49%
GlyCCC	44%	5%	15%
LeuCAG	12% (8% + 4%)	4%	35%
LeuTAA	17%	9%	23%
SerACT	10%	2%	55%
SerGCT	16% (9% + 7%)	5%	20%
ThrCGT	24%	12%	35%
TyrGTA	7% (4% + 3%)	3%	59%
ValCAC	18%	10%	23%

Table S2: Sequences used for the design of custom Taqman small RNA assays. Between brackets are the nucleotides that were used to extend the original target sequences if the sequence length was beneath 17 nucleotides. Nucleotides in red represent single nucleotide polymorphisms.

**Target**	**Sequence**	**Length**
Arg-TCG$^{53-67}$	5’ (GG) GUUCGAAUCCCUCCG 3’	15 (17)
Gly-CCC$^{1-30}$	5’ GCAUUGGUGGUUCAGUGGUAGAAUUCUCGC 3’	30
Leu-CAG$^{53-67}$	5’ (UG) GGAGGCGUGGGUUCGAA 3’	15 (17)
Leu-CAG$^{54-69}$	5’(G) GAGGCGUGGGUUCGAA 3’	16 (17)
Leu-TAA$^{8-24}$	5’ UGGCCGAGUGGUUAAGG 3’	17
Ser-ACT$^{1-15}$	5’ (UU) GCCCGGUUAGCUCAG 3’	15 (17)
Ser-GCT$^{1-18}$	5’ GACGACGUGGCCGAGUGG 3’	18
Ser-GCT$^{1-24}$	5’ GACGACGUGGCCGAGUGGUUAAGG 3’	24
Thr-CGT$^{16-30}$	5’ (AG) CGGAAGCGUGCUGGG 3’	15 (17)
Tyr-GTA$^{9-29}$	5’ AGCUCAGUUGGUAGAGCGUGG 3’	21
Tyr-GTA$^{1-19}$	5’ CUCUCGAUAGCUCAGUUGG 3’	19
Val-CAC$^{1-32}$	5’ GUUUCCGUAGUGUAGUGGUUAUCACGUUCGCC 3’	32

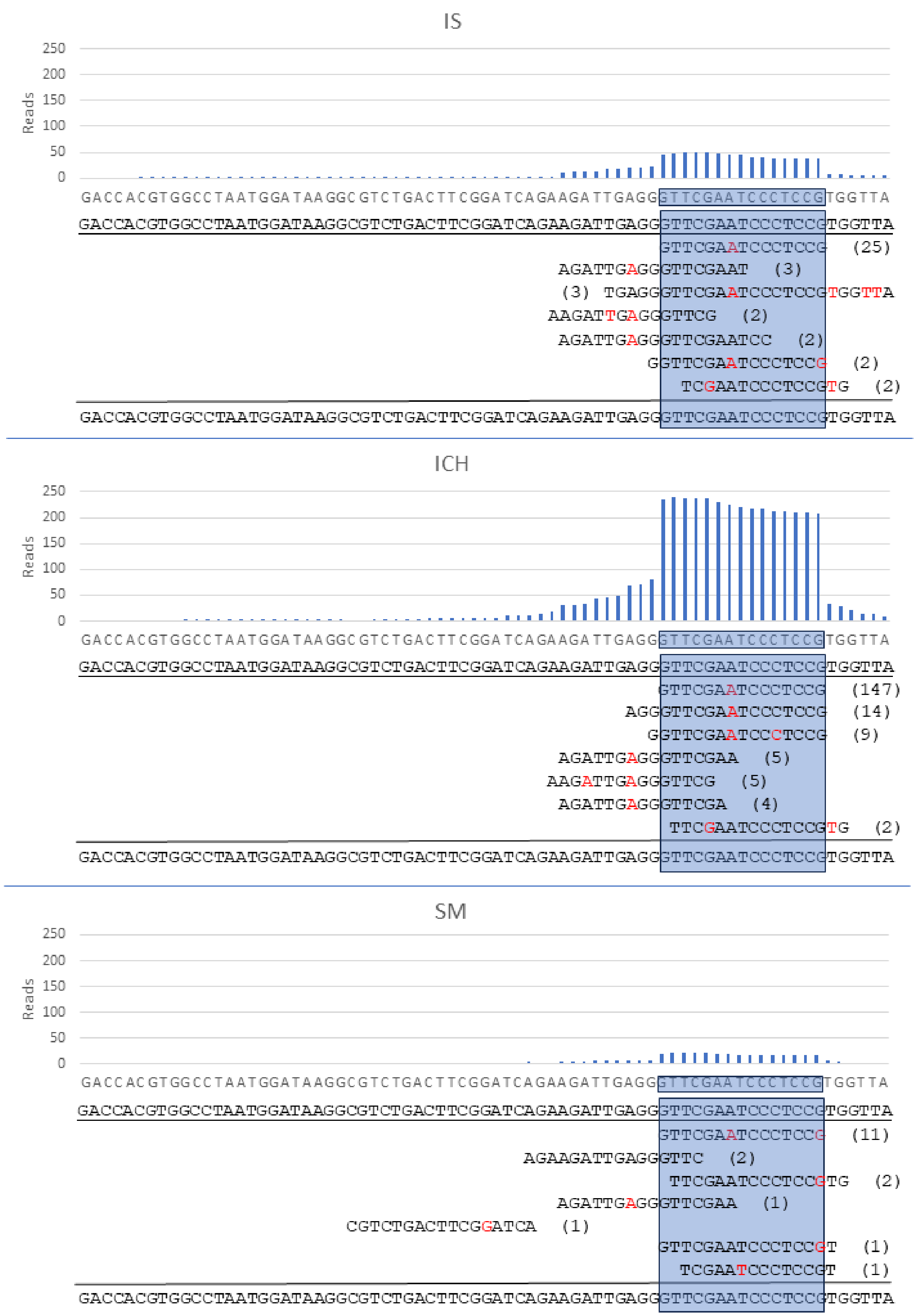

Figure S1: Read density of fragments derived from **ArgTCG** (that were mapped to chr6trna4) in ischemic stroke (IS), intracerebral hemorrhage (ICH) and stroke mimic (SM) patients. The seven most abundant sequences from the RNA sequencing data are displayed with the total amount of reads of that specific sequence between brackets. Indicated in red are the nucleotides that were substituted with a different nucleotide in at least 10% of the reads.

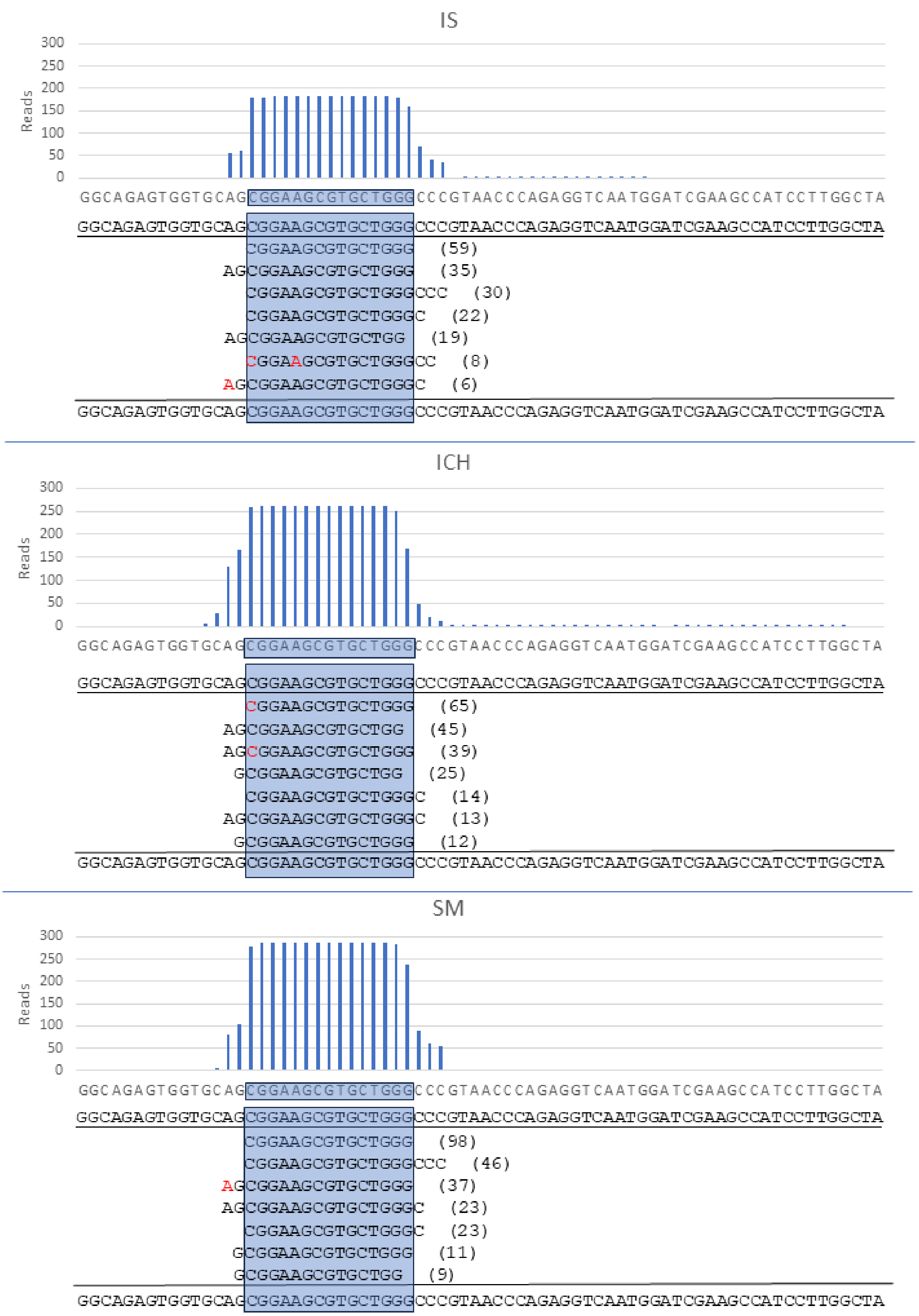

Figure S2: Read density of fragments derived from **ThrCGT** (that were mapped to chr6trna151) in ischemic stroke (IS), intracerebral hemorrhage (ICH) and stroke mimic (SM) patients. The seven most abundant sequences from the RNA sequencing data are displayed with the total amount of reads of that specific sequence between brackets. Indicated in red are the nucleotides that were substituted with a different nucleotide in at least 10% of the reads.

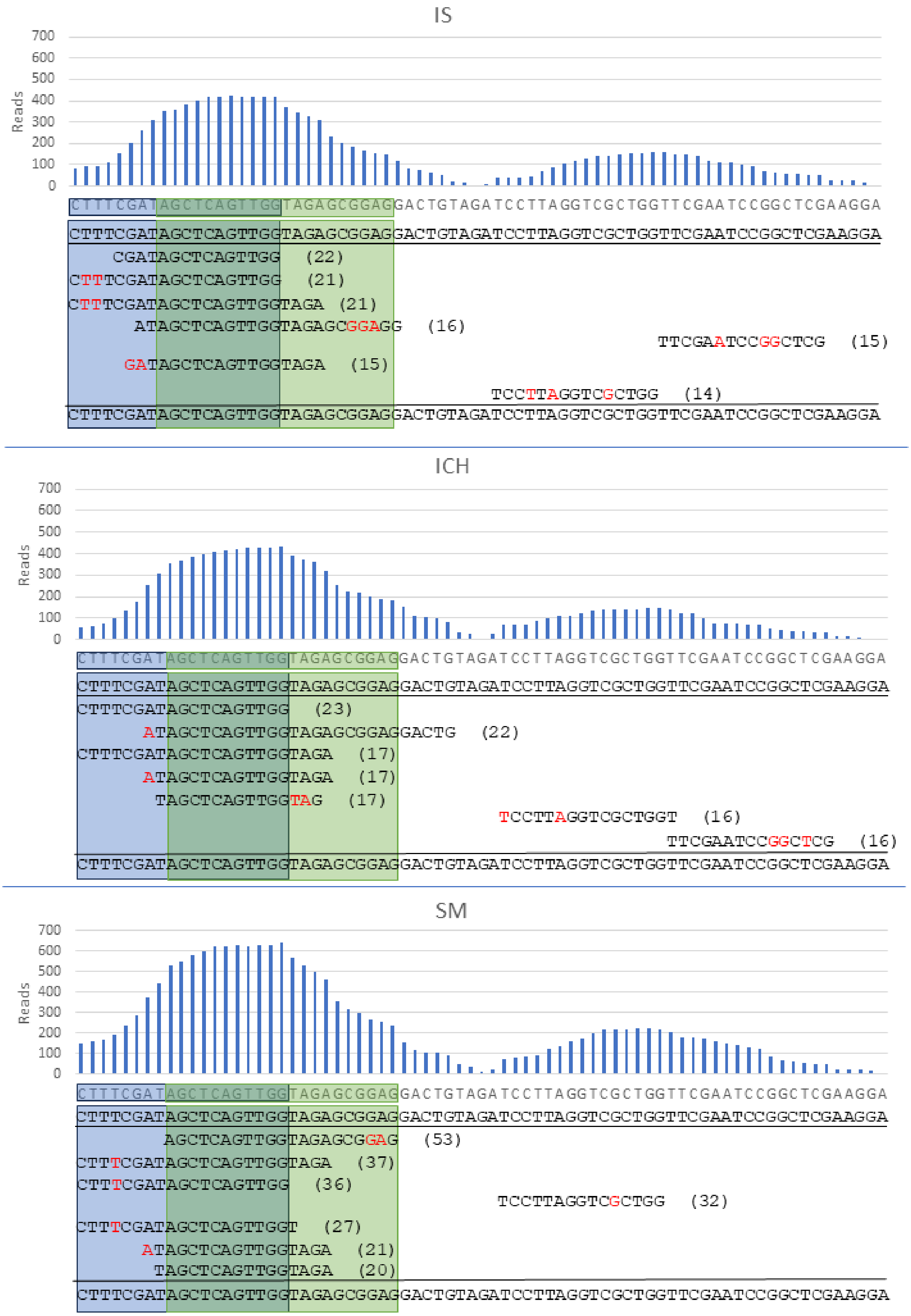

Figure S3: Read density of fragments derived from **TyrGTA** (that were mapped to chr6trna15) in ischemic stroke (IS), intracerebral hemorrhage (ICH) and stroke mimic (SM) patients. The seven most abundant sequences from the RNA sequencing data are displayed with the total amount of reads of that specific sequence between brackets. Indicated in red are the nucleotides that were substituted with a different nucleotide in at least 10% of the reads.

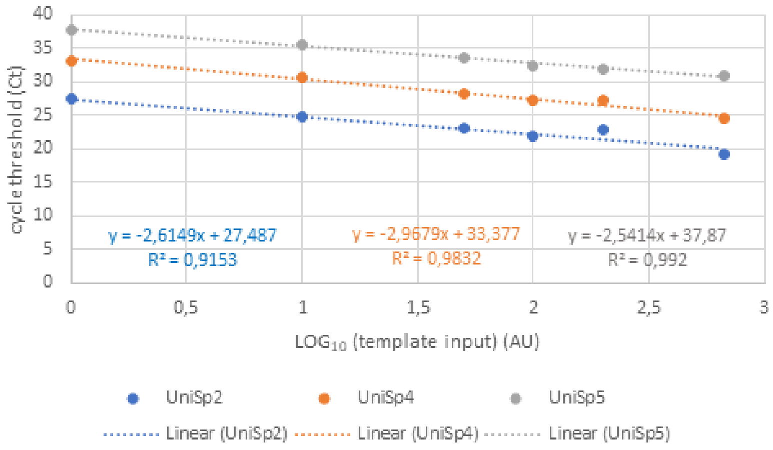

Efficiency:	
UniSp2	2,412275
UniSp4	2,172383
UniSp5	2,474498

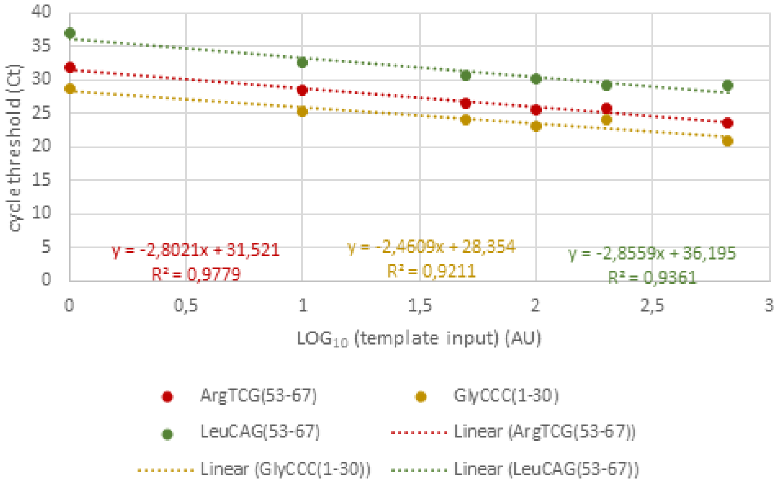

Efficiency:	
ArgTCG$^{53-67}$	2,274442
GlyCCC$^{1-30}$	2,548877
LeuCAG$^{53-67}$	2,239535

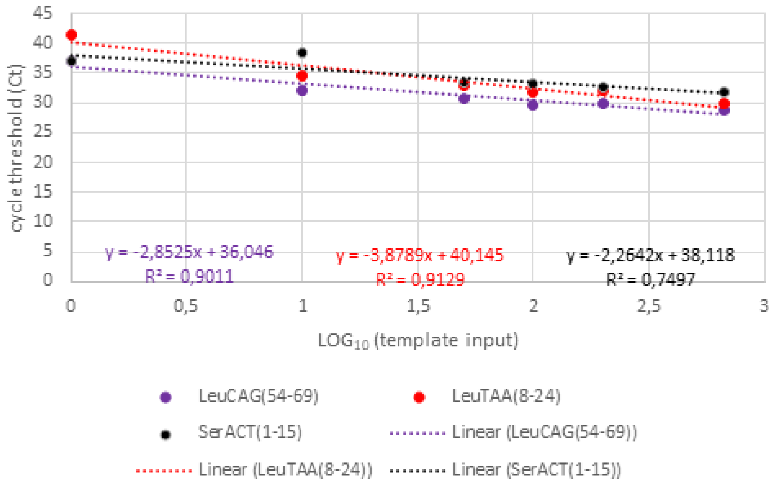

Efficiency:	
LeuCAG$^{54-69}$	2,241679
LeuTAA$^{8-24}$	1,810526
SerACT$^{1-15}$	2,764817

Figure S4: Characterization of qPCR efficiencies of Custom Taqman Small RNA assays for UniSp2, UniSp4, UniSp5, ArgTCG$^{53-67}$, GlyCCC$^{1-30}$, LeuCAG$^{53-67}$, LeuCAG$^{54-69}$, LeuTAA$^{8-24}$, and SerACT$^{1-15}$ by serial dilution of pooled cDNA of patient plasma samples. Cycle threshold was plotted versus the logarithmic function of the relative input. The equation: efficiency = $10^{(-1/slope)}$ was used to calculate the corresponding real-time PCR efficiencies[1].

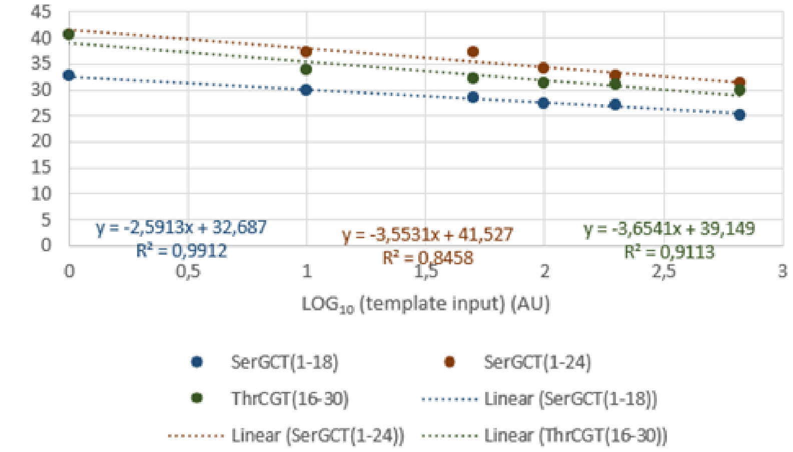

	Efficiency:
SerGCT1	2,431663
SerGCT2	1,911812
ThrCGT	1,877877

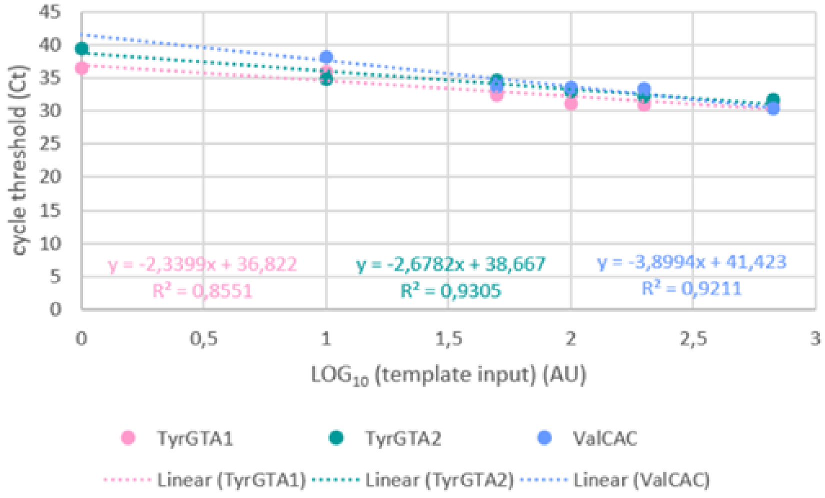

	Efficiency:
TyrGTA1	2,675326
TyrGTA2	2,362552
ValCAC	1,804893

Figure S5: Characterization of qPCR efficiencies of Custom Taqman Small RNA assays for $SerGCT^{1\text{-}18}$, $SerGCT^{1\text{-}24}$, $ThrCGT^{16\text{-}30}$, $TyrGTA^{9\text{-}29}$, $TyrGTA^{1\text{-}19}$, and $ValCAC^{1\text{-}32}$ by serial dilution of pooled cDNA of patient plasma samples. Cycle threshold was plotted versus the logarithmic function of the relative input. The equation: efficiency = $10^{(-1/slope)}$ was used to calculate the corresponding real-time PCR efficiencies[1].

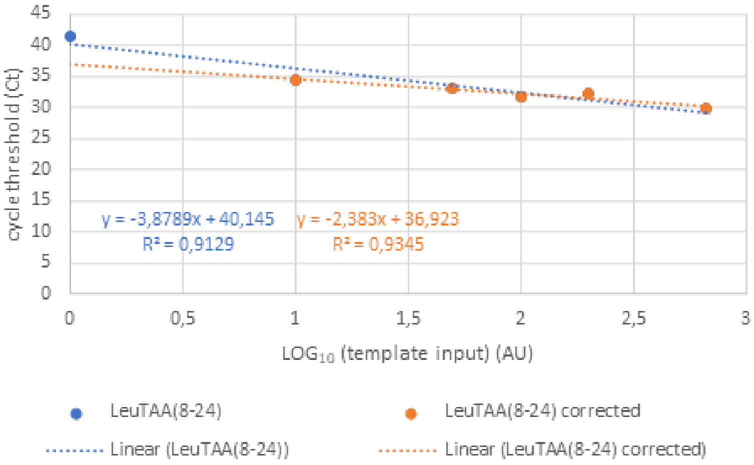

	Efficiency:
LeuTAA8-24	1,810526
LeuTAA8-24 corrected	2,628083

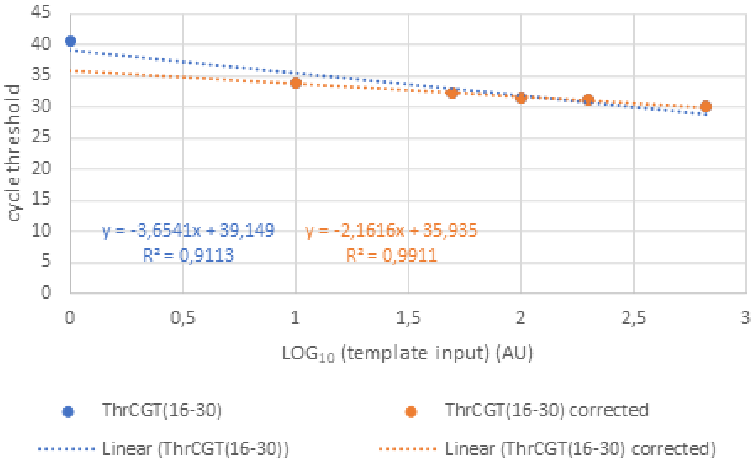

	Efficiency:
ThrCGT16-30	1,877877
ThrCGT16-30 corrected	2,901485

Figure S6: Characterization of qPCR efficiencies of Custom Taqman Small RNA assays for LeuTAA$^{8-24}$ and ThrCGT$^{16-30}$ by serial dilution of pooled cDNA of patient plasma samples with and without correction for the highest dilution. Cycle threshold was plotted versus the logarithmic function of the relative input. The equation: efficiency = $10^{(-1/slope)}$ was used to calculate the corresponding real-time PCR efficiencies[1].

References:

1. Pfaffl, M.W. (2001). A new mathematical model for relative quantification in real-time PCR. Nucleic Acids Res *29*, 6. 10.1093/nar/29.9.e45.
