# Supplementary material for: Validation of tRNA-derived fragments as diagnostic biomarkers in suspected acute stroke; limitations in analysis and quantification methods
Source: Mol Ther Nucleic Acids. 2025 May 5;36(2):102553. doi: 10.1016/j.omtn.2025.102553 (PMC12143625; doi:10.1016/j.omtn.2025.102553)
Supplement: Document S2. Article plus supplemental information [file mmc2.pdf]

Molecular Therapy
Nucleic Acids
Original Article

Validation of tRNA-derived fragments as diagnostic biomarkers in suspected acute stroke; limitations in analysis and quantification methods

Tamar Woudenberg,[1,2] M. Leontien van der Bent,[1,2] Daphne A.L. van den Homberg,[1,2] T. Truc My Nguyen,[3] Marieke J.H. Wermer,[3,4] Ido R. van den Wijngaard,[3,5,6] Paul H.A. Quax,[1,2] A. Yaël Nossent,[7,8] and Nyika D. Kruyt[3,5,8]

[1]Department of Vascular Surgery, Leiden University Medical Center, 2300 RC Leiden, the Netherlands; [2]Einthoven Laboratory for Experimental Vascular Medicine, Leiden University Medical Center, 2300 RC Leiden, the Netherlands; [3]Department of Neurology, Leiden University Medical Center, 2300 RC Leiden, the Netherlands; [4]Department of Neurology, University Medical Center Groningen, 9700 RB Groningen, the Netherlands; [5]University Neurovascular Center Leiden-The Hague, 2300 RC Leiden, the Netherlands; [6]Neurology, Haaglanden Medical Center, 2501 CK The Hague, the Netherlands; [7]Department of Nutrition, Exercise and Sports, University of Copenhagen, 2200 Copenhagen, Denmark

In acute stroke, timely diagnosis is essential to prevent extensive neuronal damage and improve patient outcomes. However, differentiating between ischemic stroke, intracerebral hemorrhage, and stroke mimics remains challenging. Transfer RNA-derived fragments (tRFs) have emerged as potential biomarkers for distinguishing between these stroke subtypes. Here we used reverse transcription-quantitative PCR (RT-qPCR) to investigate the expression of specific tRFs that we previously identified in small RNA sequencing data as potential biomarkers. Out of 12 measured tRFs, only the fragments ArgTCG$^{53-67}$ and TyrGTA$^{1-19}$ showed a trend for differential expression between stroke subtypes, but with insufficient predictive value to be of use in clinical practice. Combining expression data of specific tRFs into a joint model did not improve the predictive ability. Technical and computational challenges may compromise the reliability of tRF expression data from RNA sequencing, possibly explaining our inability to validate the specific tRFs as potential stroke biomarkers. Moreover, the limitations of RT-qPCR challenge a reliable quantification of these fragments even further. Our findings highlight the need for improved quantitative methods for tRF analysis to fully exploit their potential as clinically meaningful biomarkers. Addressing these technical barriers could unlock the diagnostic potential of tRFs, facilitating faster, more accurate stroke subtype identification in an acute setting.

INTRODUCTION

In acute stroke, millions of neurons die each minute, making it pivotal to initiate treatment as soon as possible to minimize brain damage and improve patient outcomes.[1,2] The appropriate intervention depends on the stroke subtype, with ischemic stroke (IS) and intracerebral hemorrhage (ICH) requiring different treatments. For IS, intravenous thrombolysis (IVT) or endovascular thrombectomy (EVT) is commonly employed, with both demonstrating efficacy.[2,3] However, their success diminishes with time, as the persisting stroke causes irreparable damage. Therefore, rapid and accurate diagnosis is critical. While neuroimaging—particularly computed tomography (CT) scanning—is effective in ruling out ICH in acute settings, its sensitivity for detecting IS is limited. Moreover, up to 40% of patients suspected to have a stroke by emergency services are ultimately diagnosed with a stroke mimic (SM), where symptoms arise from a non-stroke pathology.[4–6] Even after hospital evaluation and neuroimaging, up to 20% of patients treated with IVT are later identified as stroke mimics.[7] A reliable biomarker, readily detectable early after symptom onset, that accurately distinguishes between IS, ICH, and SM would thus be of significant clinical value.

Unencapsulated small noncoding RNAs (sncRNAs) hold great potential as biomarkers, as sncRNAs are released rapidly upon cellular stress and remain stable in the circulation due to their association with lipoproteins or RNA-binding proteins.[8,9] Moreover, expression profiles of microRNAs, a class of sncRNA, appear to change in response to acute stroke.[10,11] A more recently discovered class of sncRNA are transfer RNA-derived fragments (tRFs) which can be detected with small RNA sequencing.[12,13] The production of tRFs is evolutionarily conserved,[14] and tRF expression exhibits disease specificity.[15,16] They are recognized as biologically active

Received 17 December 2024; accepted 29 April 2025;
https://doi.org/10.1016/j.omtn.2025.102553.

[8]These authors contributed equally

Correspondence: A. Yaël Nossent, Department of Nutrition, Exercise and Sports, University of Copenhagen, 2200 Copenhagen, Denmark.
E-mail: nossent@nexs.ku.dk
Correspondence: Nyika D. Kruyt, Department of Neurology, Leiden University Medical Center, 2300 RC Leiden, the Netherlands.
E-mail: n.d.kruyt@lumc.nl

© 2025 The Author(s).
Published by Elsevier Inc. on behalf of The American Society of Gene and Cell Therapy.
This is an open access article under the CC BY license (http://creativecommons.org/licenses/by/4.0/).

molecules involved in the regulation of gene expression,[17] highlighting them as promising biomarker candidates.

In a pilot study, we demonstrated that combining the expression data of a small number of circulating tRFs can distinguish IS from ICH or SM with high accuracy.[13] These findings were confirmed in a publicly available small RNA sequencing dataset from a different study population.[18] However, the sample size was small and the available dataset for verification did not include patients with ICH or SM, but included only healthy control subjects. Moreover, the RNA sequencing detection methodology for quantification of tRFs has limitations, due to mapping ambiguity and reverse transcription interference.[19] Therefore, further validation with an independent detection method was required. For this study, our aim was to validate the findings from this pilot study in a larger, independent cohort, including IS, ICH, and SM patients, using reverse transcription-quantitative PCR (RT-qPCR) as an independent detection method (the gold standard for validation of RNA sequencing data). To improve the potential clinical applicability of tRF-based biomarkers, the pilot study and this study specifically analyzed extracellular vesicle (EV)-depleted plasma, ensuring that only non-vesicular RNA fractions were assessed. This choice was motivated by two key considerations. First, we aimed to capture RNA species released due to acute tissue damage, as opposed to those actively secreted in EVs, which may reflect more regulated processes occurring later after stroke onset. Second, non-vesicular RNA is more accessible for rapid point-of-care testing, simplifying potential clinical implementation.

RESULTS

Patient characteristics

We included 87 patients suspected by paramedics to have an acute stroke in the last 6 h. After clinical work up, 34 patients had IS, 25 patients had ICH, and 28 patients had an SM (Table 1). The mean age was 68 ± 14 years, and median onset-to-arrival at the emergency department was 73 min (interquartile range [IQR]: 46–134 min).

The stroke severity in IS patients was mild with a median National Institute of Health Stroke Scale (NIHSS) score of 3 (IQR: 2–7), and in ICH patients, it was moderate with a median NIHSS score of 9 (IQR: 5–15). The SM group had a median NIHSS score of 0 (IQR: 0–4). In the IS group, 10 patients (29.4%) showed a proximal occlusion on CT angiography imaging. In 12 other patients (35.3%), additional magnetic resonance imaging (MRI) imaging confirmed the IS diagnosis. All patients received CT perfusion imaging, and 8 patients with an SM (29%) received additional MRI to decrease the likelihood of IS being the underlying pathological condition. The most common causes for SM were peripheral vestibular syndrome (36%), seizure (32%), and functional neurological disorders (11%).

tRF length and origin

RT-qPCR is a targeted method, requiring prior knowledge of target sequences. For effective validation, we first determined our target sequences from the original RNA sequencing data. Our pilot study highlighted the sum of fragments derived from tRNAs Arg-TCG, Gly-CCC, Leu-CAG, Leu-TAA, Ser-ACT, Ser-GCT, Thr-CGT, Tyr-GTA, and Val-CAC as potential biomarkers.[13] Assuming that the most prevalent fragment derived from these tRNAs made the largest contribution to the diagnostic predictions, we aimed to select a single fragment for each tRNA parent with the highest number of reads from the original RNA sequencing data as target sequence for our RT-qPCR analysis. For six tRNAs, a single most abundant fragment was selected (Figure 1). Arg-TCG and Gly-CCC showed a sole dominant fragment, where the fragment with the highest number of reads accounted for 51% and 44% of the total amount of reads attributed to Arg-TCG and Gly-CCC, respectively (Table S1). For Leu-TAA, Ser-ACT, Thr-CGT, and Val-CAC, the most dominant fragment was selected as a target sequence. Reads mapped to Leu-CAG, Ser-GCT, and Tyr-GTA showed several fragments with similar high abundance. For these tRNAs, the two fragments with the highest number of reads were selected (Figures 1C, 1F, and 1H; Table S1). Rare fragments (reads only occurring in five samples or less) also contributed substantially to the total read count. The most abundant fragments were derived from various regions of the parent tRNAs. Although seven dominant fragments were derived from the 5′ arm, they varied in both starting point and length, with the shortest measuring 15 nucleotides (SerACT$^{1-15}$ and ThrCGT$^{16-30}$; Figures 1E and 1G) and the longest extending to 32 nucleotides (ValCAC$^{1-32}$; Figure 1I). Fragments derived from ArgTCG (Figure 1A) and LeuCAG (Figure 1C) originated from the 3′ arm. While ArgTCG$^{53-67}$ and LeuCAG$^{53-67}$ showed the same starting point and length, LeuCAG$^{54-69}$ deviated in length and starting point. Even though researchers have tried to classify tRFs according to their region of origin in their respective parent full-length tRNAs, our findings indicated that no specific region of a mature tRNA is preferentially processed or escapes degradation.

tRF expression in suspected stroke patients

Expression levels of these dominant fragments were determined in EV-depleted plasma of the suspected stroke code patients using RT-qPCR. Most of the fragments appeared to have similar expression patterns in plasma of IS, ICH, and SM patients (Figure 2). The elevation of tRF-TyrGTA$^{1-19}$ levels in plasma of SM patients compared to ICH patients almost reached significance (p = 0.0545), and a trend for elevated tRF-TyrGTA$^{1-19}$ levels was observed for IS patients when compared to ICH patients (p = 0.1041). Other trends for differential, albeit not statistically different, expression were observed in tRF-ArgTCG$^{53-67}$ levels (IS vs. ICH; p = 0.0705, IS vs. SM; p = 0.0808).

Common tRF model

In our pilot study, the identified tRNA fragment "hits" were validated in an independent cohort,[18] and a "common tRF model" was composed: a combination of three isodecoders (different tRNA genes that share the same anticodon) that showed high diagnostic accuracy in distinguishing IS patients from ICH and SM patients. This model consisted of the isodecoders tRNA-TyrGTA, tRNA-ThrCGT, and tRNA-ValCAC. We assessed the performance of this model by

Table 1. Patient baseline characteristics of the investigated cohort

	Total no. of patients (n = 87)	Ischemic stroke (n = 34)	Intracerebral hemorrhage (n = 25)	Stroke mimic (n = 28)
Age, years, mean (SD)	68 (14)	71 (15)	71 (11)	63 (14)
Male sex, n (%)	42 (48)	19 (56)	15 (60)	8 (29)
Medical history, n (%)				
Ischemic stroke/TIA[a]	24 (28)	10 (29)	7 (28)	7 (25)
Intracerebral hemorrhage	5 (6)	2 (6)	1 (4)	2 (7)
Atrial fibrillation	14 (16)	6 (18)	5 (20)	3 (11)
Diabetes mellitus	14 (16)	7 (21)	2 (8)	5 (18)
Myocardial infarction	12 (14)	5 (15)	4 (16)	3 (11)
Hyperlipidemia	40 (46)	18 (53)	11 (44)	11 (39)
Hypertension	45 (52)	22 (65)	10 (40)	13 (46)
Medication use, n (%)				
Oral anticoagulation	16 (18)	6 (18)	5 (20)	5 (18)
Platelet aggregation inhibitors	32 (37)	15 (44)	8 (32)	9 (32)
Hospital admission				
Onset-to-arrival time, median (IQR) in minutes	73 (46–134)	69 (46–117)	59 (45–105)	90 (47–175)
NIHSS[b] score	4 (2–8)	3 (2–7)	9 (5–15)	0 (0–4)
Neuro-imaging				
Stroke lesion confirmed, n (%)	–	22 (65)	25 (100)	–
Ischemic lesion excluded with MRI, n (%)	–	–	–	8 (29)
Reperfusion therapy				
Intravenous thrombolysis, n (%)	–	25 (74)	–	1 (4)
Endovascular thrombectomy, n (%)	–	10 (29)	–	–
Discharge location, n (%)				
Home	40 (46)	15 (44)	0 (0)	25 (89)
Other hospital	11 (13)	3 (9)	6 (24)	2 (7)
Nursing home	4 (5)	2 (6)	2 (8)	0 (0)
Rehabilitation center	21 (24)	9 (27)	11 (44)	1 (3)
Deceased	6 (7)	2 (6)	4 (16)	0 (0)
mRS after 90 days median (IQR)	3 (2–3)	2 (2–3)	3 (2–5)	–
Three most common causes for SM				
Peripheral vestibular syndrome, n (%)	–	–	–	10 (36)
Seizure, n (%)	–	–	–	9 (32)
Functional neurological disorder, n (%)	–	–	–	3 (11)

[a]TIA, transient ischemic attack.
[b]NIHSS, National Institute of Health Stroke Scale.

looking at the expression of TyrGTA$^{1-19}$, TyrGTA$^{9-29}$, ThrCGT$^{16-30}$, and ValCAC$^{1-32}$, and their combination, in the plasma samples of our current cohort. In distinguishing SM patients from stroke patients (SM vs. IS and ICH), the common tRF model showed a higher area under the receiver operating characteristic (ROC) curve (AUC) (0.577; 95% confidence interval [CI]: 0.447–0.706) than the individual fragments (Figure 3). However, when discriminating either IS or ICH patients from the other two remaining groups (IS vs. ICH and SM or ICH vs. IS and SM), the AUCs of some individual fragments were numerically higher than that of the common tRF model, although their CIs overlapped. For IS, the diagnostic accuracies of ValCAC$^{1-32}$ (AUC: 0.560; 95% CI: 0.424–0.686), TyrGTA$^{1-19}$ (AUC: 0.574; 95% CI: 0.444–0.705), and ThrCGT$^{16-30}$ (AUC: 0.552; 95% CI: 0.428–0.675) were comparable to that of the common tRF model (AUC: 0.544; 95% CI: 0.413–0.666). Similarly, for ICH, the AUC of the common tRF model (AUC: 0.371; 95% CI: 0.238–0.504) was within the CIs of ValCAC$^{1-32}$ (AUC: 0.412; 95% CI: 0.282–0.542), TyrGTA$^{1-19}$ (AUC: 0.485; 95% CI: 0.359–0.611), and ThrCGT$^{16-30}$ (AUC: 0.484; 95% CI: 0.350–0.619). From all of the individual fragments in the common tRF model, TyrGTA$^{1-19}$ had the

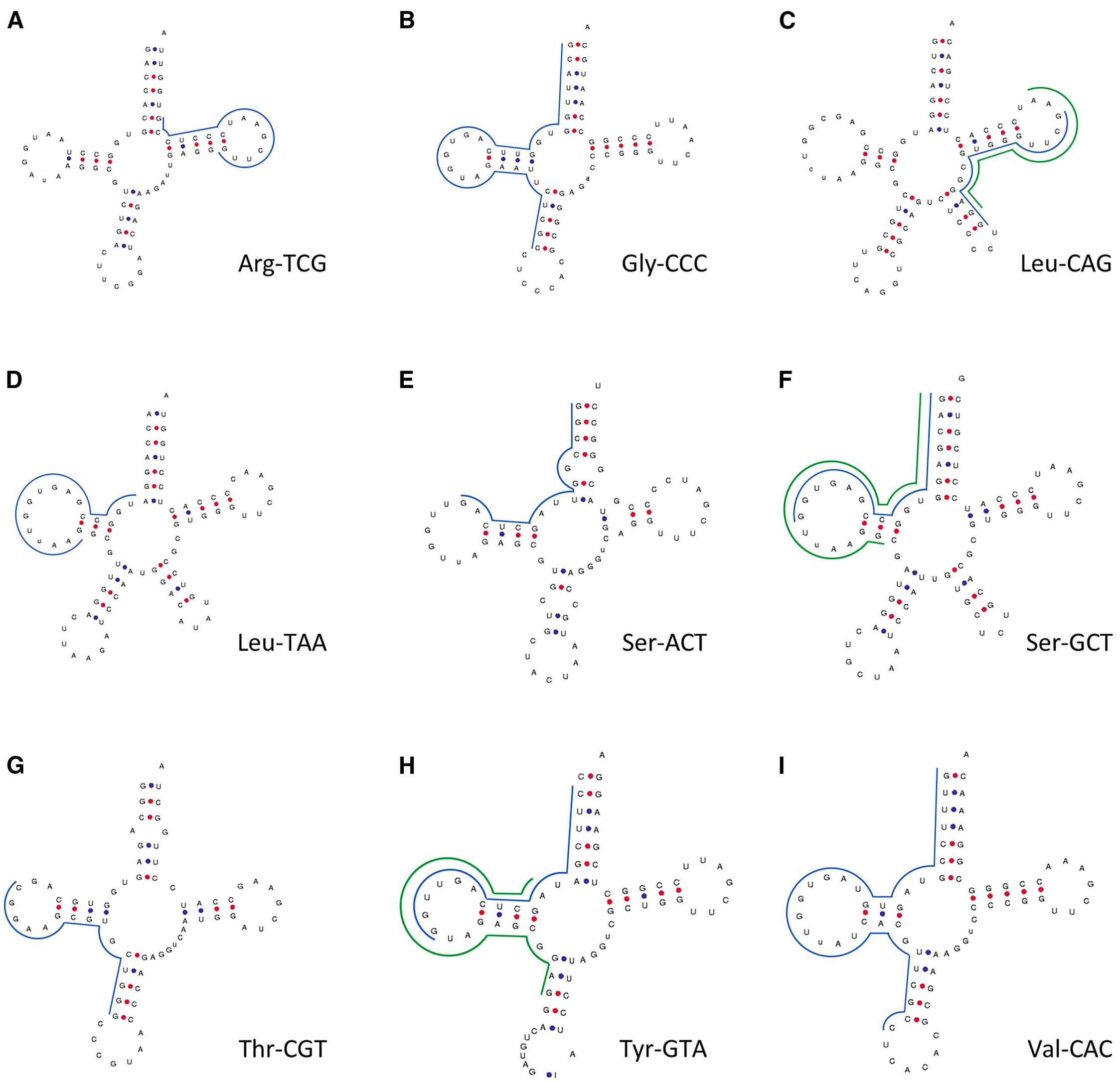

Figure 1. Mature target tRNAs with their selected tRNA-derived fragments

(A–I) Secondary structures of arginin-TCG (A), glycin-CCC (B), leucin-CAG (C), leucin-TAA (D), serin-ACT (E), serin-GCT (F), threonin-CGT (G), tyrosin-GTA (H), and valin-CAC (I), and the location of their representative selected fragments. The secondary structures of the mature tRNAs were derived from GtRNAdb.

highest accuracy in discriminating IS patients (AUC: 0.574; 95% CI: 0.444–0.705) and SM patients (AUC: 0.555; 95% CI: 0.428–0.682), and TyrGTA$^{9-29}$ had the highest diagnostic accuracy in discriminating ICH patients (AUC: 0.485; 95% CI: 0.359–0.611).

Validation of regression analysis

As in the pilot study, we performed a least absolute shrinkage and selection operator (LASSO) regression analysis on our expression data to identify an optimal model to predict the outcome in our cohort. The LASSO analysis showed that the lowest cross-validated error was obtained with just one biomarker in the model TyrGTA$^{1-19}$, indicating that there was no added diagnostic value in combining tRFs into profiles to predict outcome in this cohort.

DISCUSSION

We were not able to reproduce findings from a pilot study in the current study with a larger, independent cohort of patients, using RT-qPCR, the gold-standard method for validation of RNA sequencing data. In this setting, the previously defined model held insufficient predictive value to be of use in clinical practice.

We were able to detect and quantify all selected fragments via RT-qPCR. In our pilot study, the fragments from these isodecoders showed high diagnostic accuracy for distinguishing IS from ICH and SM patients, which was expected given that validation of hits in the pilot study was performed in a cohort of IS patients and healthy controls, lacking ICH and SM patients.[13,18] However, despite

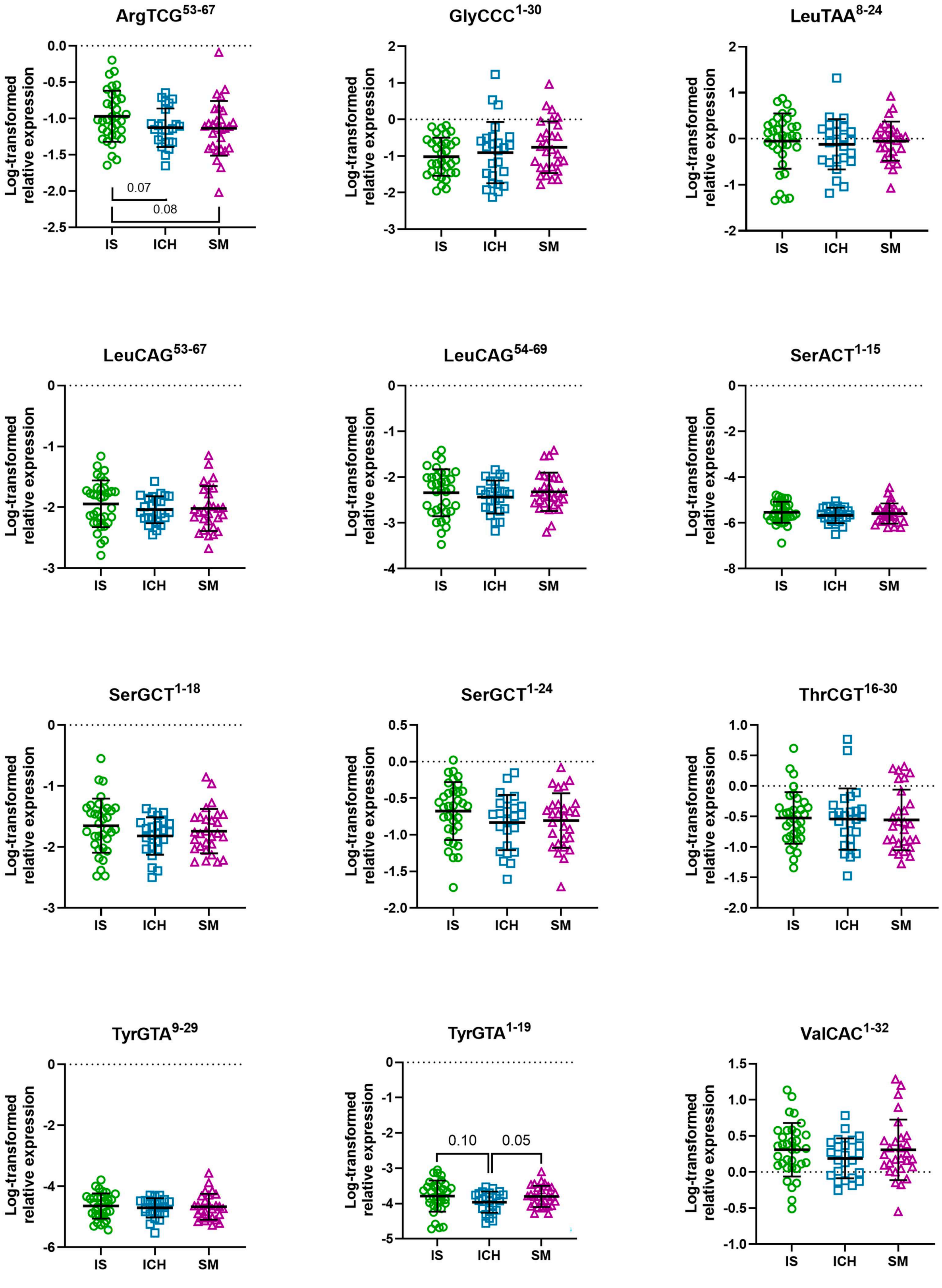

(legend on next page)

the promising RNA sequencing results, the investigated fragments, either individually or in combination, demonstrated insufficient diagnostic value in the current study.

While in our pilot study the sum of all fragments per isodecoder were considered in the analysis, RT-qPCR focuses on the expression levels of individual sequences. In taking an individual sequence as a representative, we assumed that the contribution of the remaining fragments is negligible. When looking at the original RNA sequencing data, this seems to be the case for Arg-TCG and Gly-CCC, where most of the reads are attributed to ArgTCG$^{53–67}$ and GlyCCC$^{1–30}$ (Table S1; Figure S1). However, for other tRNAs, the prominence of a single most abundant fragment was less distinct. While ThrCGT$^{16–30}$ showed a sole fragment as most abundant, other abundant fragments derived from Thr-CGT still contribute substantially to the total sum of fragments derived from Thr-CGT (Table S1). Interestingly, other highly contributing fragments originated from the same region within the full-length sequence of the mature tRNA-Thr-CGT (Figure S2). Even though the prominence of a single most abundant fragment was the least distinct for the Tyr-GTA (Table S1; Figure S3), we observed a trend for differential expression in one of the selected fragments. Therefore, while the contribution of other fragments may partially explain the differences in diagnostic accuracies between the two studies, the observed trends for differential expression in ArgTCG and TyrGTA—despite one having a distinct dominant fragment and the other not—suggest that other factors are at play as well.

The computational difficulties of analyzing tRF expression from small RNA sequencing data are a possible contributing factor to the differences in outcome. In RNA sequencing, reads are mapped to the respective genes of origin. However, the human genome contains over 600 tRNA genes, distributed across multiple chromosomal loci, with many tRNA isotypes and isoacceptors having nearly identical sequences. Consequently, a single read associated with a tRF can be mapped to multiple loci. The multimapping can lead to an overestimation of expression, but can be controlled by various strategies such as assigning multimapping reads to a locus with the fewest mismatches, distributing the counts of multimapping reads across all loci, or discarding multimapping reads entirely. However, all approaches have the potential to lead to either under- or overestimation of expression, and risk losing critical information.[19–21] While locus-specific expression may not always be essential in identifying tRFs as biomarkers, neglecting this information could still obscure unique expression patterns that contribute to biomarker specificity or reflect underlying biology, potentially impacting diagnostic accuracy.[22]

Moreover, tRNAs undergo extensive post-transcriptional modifications,[23] which could have influenced both the RNA sequencing results from our pilot study and the RT-qPCR validation in the current study. Post-transcriptional modifications can interfere with reverse transcription, leading to misincorporations or amplification biases.[19,24] Given that tRNA modifications are often preserved in tRFs, this could have led to an underestimation of tRF expression in both the pilot study and the current study. While a comparative analysis of known modifications within the measured fragments is beyond the scope of this study, future research should investigate their potential impact on detection methodologies, as post-transcriptional modifications are increasingly recognized in various RNA species and may even exhibit disease-specific patterns.[25–27]

Furthermore, small RNA sequencing relies on adapter ligation, which requires a 5′-phosphate group and a 3′-hydroxyl group. However, tRFs carry chemical modifications at their termini that can hinder ligation efficiency, leading to biases in detection.[28–30] This issue particularly affects 3′-arm-derived tRFs (3′-tRFs), which are inefficiently captured by conventional ligation methods.[31] In our pilot RNA sequencing data, most dominant fragments originated from the 5′-arm, while read densities at the 3′-arm for TyrGTA and ThrCGT were markedly lower (Figures S2 and S3). This suggests that 3′-tRFs may have been systematically underrepresented in the pilot study, potentially affecting the accuracy of initial biomarker selection.

Accurately quantifying tRF expression through RT-qPCR remains a technical challenge as well. The exceptionally short length of many fragments complicates the design of Taqman qPCR kits, which require a minimum length of 17 nucleotides. Variability in the precise ends of these fragments can compromise PCR assay specificity, potentially leading to non-specific amplification and artificially high PCR efficiencies. This effect is particularly relevant for short tRF sequences, where minor mismatches or incomplete primer binding may result in increased amplification rates. Indeed, we observed high PCR efficiencies in our assays (Figures S4 and S5). For two targets, Leu-TAA$^{8–24}$ and Thr-CGT$^{16–30}$, the highest dilution points were detected beyond 40 cycles, indicating a gray detection area with limited precision. While we retained these points for consistency, excluding them would further increase PCR efficiencies (Leu-TAA$^{8–24}$: 1.81 $\rightarrow$ 2.63; Thr-CGT$^{16–30}$: 1.88 $\rightarrow$ 2.90) (Figure S6). This highlights the challenge of accurately quantifying low-abundant tRFs, with assays being impacted by sequence constraints and post-transcriptional modifications.

Therefore, reliable quantification methods for assessing tRF expression levels in biological samples is imperative to confidently reject or accept these fragments as potential biomarkers for stroke subtypes. Alternative approaches, like microarrays, may provide an opportunity to measure tRFs with great specificity and high throughput, enabling large-scale screening of tRFs.[32–35] However, this might

Figure 2. Log-transformed expression patterns of tRNA-derived fragments in extracellular vesicle-poor plasma of patients with different stroke subtypes
Data were normalized to spike-ins UniSp2, UniSp4, and UniSp5 and log-transformed. IS, ischemic stroke; ICH, intracerebral hemorrhage; SM, stroke mimic. Data are represented as mean ± SD.

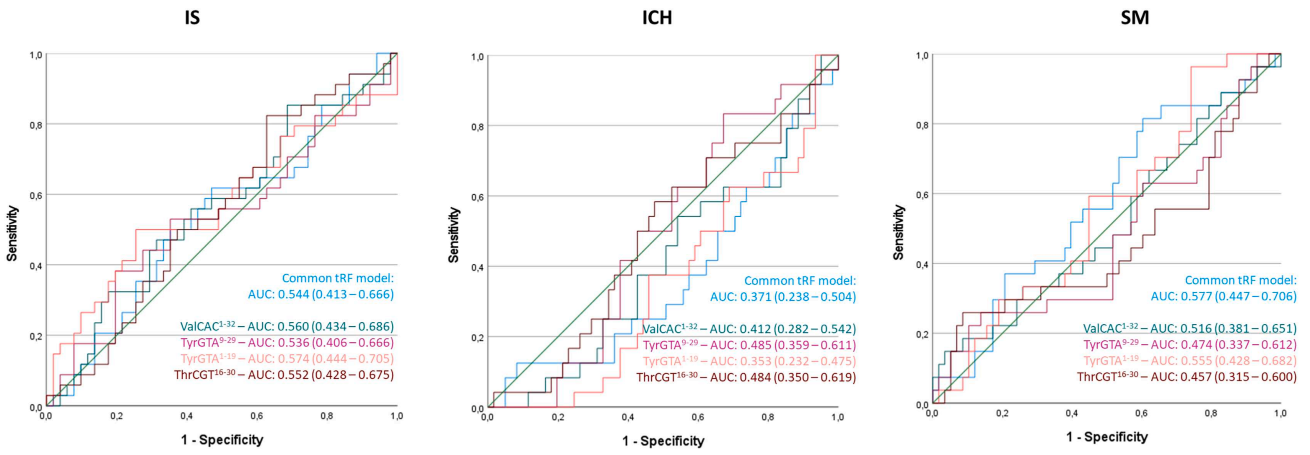

Figure 3. Performance of the common tRF model in the investigated patient cohort

ROC analysis of diagnostic accuracy of the common tRF model, and the individual tRFs, in distinguishing each patient group from the other two. Data are expressed as AUC (95% confidence interval). AUC, area under the curve; IS, ischemic stroke; ICH, intracerebral hemorrhage; SM, stroke mimic.

also be hampered by RNA modifications as they can interfere with base-pairing, and therefore hybridization efficiency.[36,37] Another promising technique is single molecule array, which provides exceptional sensitivity and precision in measuring small quantities of nucleic acids.[38] While these methods may improve quantification, they do not resolve the loss of information on the genomic origin of tRFs. Nonetheless, they could complement current strategies for studying tRFs.

Despite these limitations, the differences observed in TyrGTA$^{1-19}$ and ArgTCG$^{53-67}$ expression levels between stroke subtypes reached near-significance in our study. If technical challenges in tRF quantification can be addressed, these differences may become more pronounced, strengthening their potential as diagnostic biomarkers. In the diagnosis of stroke, it is essential to assess these fragments quickly and accurately in out-of-hospital settings, such as ambulances or general practitioner offices. To this end, we deliberately selected EV-depleted plasma as our sample source, to ensure identification of readily detectable, non-encapsulated circulating tRFs, enhancing and simplifying its usability for clinical application. For instance, point-of-care devices based on sequence complementarity can facilitate the sensitive and specific detection of tRFs, as demonstrated by the development of a detection system for epilepsy-associated tRFs.[39] Additionally, the feasibility of integrating multiple oligonucleotides into a unified signaling system has been demonstrated, paving the way for simultaneous detection of several tRFs.[40] This approach could enhance diagnostic accuracy, allowing the use of tRF profiles rather than individual tRFs as biomarkers.

In conclusion, we could not replicate our previous pilot findings in a larger validation cohort, which could be caused by technical limitations in the currently available analysis and quantification methods. However, the trends in differential expression levels of TyrGTA$^{1-19}$ and ArgTCG$^{53-67}$ between stroke subtypes suggest their potential as diagnostic biomarkers in an acute stroke setting. Addressing the challenges associated with tRF detection—such as mapping ambiguities, short fragment lengths, and modification-induced biases—is essential for future biomarker validation. Developing reliable methods to quantify tRFs in biological samples is crucial. Addressing these challenges could unlock the diagnostic potential of tRFs, enabling rapid and accurate stroke diagnosis even in out-of-hospital settings.

MATERIALS AND METHODS

Study design

The MicroRNA In Acute Stroke study was a prospective observational multicenter cohort study. Patients were recruited between January 2019 and October 2021, during office hours, from the emergency departments of the University Medical Center Leiden—The Hague, including one academic and one large non-academic emergency room (ER). A deferred informed consent procedure was used for the blood sampling on the ER in order to not interfere with time-sensitive diagnostic and treatment procedures. After this, written informed consent was acquired for the use of the already sampled blood, as well as for the use and storage of clinical and radiological patient data extracted from the electronic patient records. Ethical approval for this study was obtained from the Medical-Ethical Committee Leiden-Den Haag-Delft (P18.030; NL63060.058.17, May 18, 2018).

Patients

Patients for whom a stroke code was activated by ambulance paramedics were eligible for inclusion if they met the following criteria: (1) 18 years or older; (2) symptom onset within the past 6 h; and (3) underwent CT, CT angiography, and CT perfusion upon admission. Exclusion criteria included (1) known active malignancy or (2) the administration of heparin within the past 24 h, to ensure reliable RNA detection and quantification.[41] To enhance diagnostic certainty, only IS patients with a clinically mild to severe stroke (defined as an NIHSS score >3), IS patients with a radiological evidence of

acute IS, or those who received IVT were included. Finally, IS and ICH patients who had to be admitted to the stroke unit were included.

Final diagnosis

The final diagnosis (IS, ICH, or SM) as noted by the treating neurologist after three months was used as the outcome. Because the diagnosis of IS and SM relies on excluding ICH rather than demonstrating IS or SM cause, all IS and SM diagnoses were reviewed by a stroke neurologist (N.D.K.). In case of persisting uncertainty, the patient was excluded.

Data collection

Clinical data including demographics, medical history, medication use, stroke severity at admission, acute treatment (IVT or EVT), neuro-imaging findings, and final diagnosis were obtained from electronic patient records. Stroke severity is quantified by the NIHSS Score; scores ranging from 0 to 3 are considered minor; 4–7, mild; 8–15, moderate; and scores greater than 15 are considered severe.[42] If the NIHSS score was not recorded, this would have been reconstructed by NIHSS certified research members using the neurological examination report at admission.[43]

Sample collection

Venous blood was collected in two 9 mL VACUETTE 9NC Coagulation sodium citrate tubes (Greiner Bio-One, Alphen aan den Rijn, the Netherlands) using an 18G infusion needle. Blood was drawn upon arrival at the ER, before administration of any treatment. Samples were directly handled without shaking or swinging to prevent cell lysis and platelet activation. Tubes were kept vertically at all times after careful inversion to sufficiently mix the blood with the citrate buffer. Blood samples were processed at room temperature within 60 min after venipuncture. Sodium citrate tubes were centrifuged for 15 min at 2,500× g, plasma was transferred to a 15 mL Greiner conical bottom centrifuge tube, and centrifuged once more for 15 min at 2,500× g to generate platelet-poor plasma. The plasma was divided into aliquots of 1 mL, snap frozen in liquid nitrogen and stored at −80°C, while the bottom 200 μL platelet-rich plasma was used for hemolysis monitoring by spectrophotometry.

Ultracentrifugation

Plasma was depleted of EVs by ultracentrifugation, in compliance with the sample preparation of the pilot study.[13] Plasma (2.5 mL) was thawed and diluted once with phosphate buffered saline (PBS) and was ultracentrifuged for 70 min at a speed of 12,000 rpm/17,500× g at a temperature of 4°C with maximum acceleration (0) and slow deceleration (5) in a swinging-bucket rotor (SW55 Ti) in a Beckman Coulter XE-90 instrument (Beckman Coulter, Woerden, the Netherlands). After ultracentrifugation, the upper 4.5 mL was carefully transferred into clean 15 mL Greiner conical bottom centrifuge tubes, and filled to 5 mL with PBS. The lower, large vesicle rich fraction was stored in aliquots, while the upper fraction was centrifuged again to remove small vesicles. Samples were centrifuged for an additional 70 min at 4 °C at 37,000 rpm/166,400× g using the same acceleration and deceleration settings as before. The upper 0.5 mL was collected, as well as the middle 3.5 mL and the bottom 1 mL. All fractions were aliquoted, snap frozen on dry ice, and stored at −80°C until RNA isolation.

Sequence selection for RT-qPCR

For successful RT-qPCR, exact sequences for the tRFs were required. These specific sequences were derived by identifying the most prevalent fragments for each isodecoder that was initially selected as a potential biomarker by our pilot study, using the original RNA sequencing data. The isodecoders that were initially selected included ArgTCG, GlyCCC, LeuCAG, LeuTAA, SerACT, SerGCT, ThrCGT, TyrGTA, and ValCAC. To identify the most prevalent fragments, we compiled all the reads of all sequences that were mapped to their corresponding tRNA parent genes using R. The sequences with the highest number of reads were selected for the custom design of Taqman small RNA assays (Applied Biosystems, Thermo Fisher Scientific, cat no. 4398987, Bleiswijk, the Netherlands). As the minimum sequence length for custom-design Taqman small RNA assays is 17 nucleotides, some target sequences were extended at the 5′ end with either uracil or with the next nucleotide(s) in the sequence of the parent tRNA. The sequences for the most abundant fragments derived from TyrGTA differed in two nucleotides compared to the DNA sequence, probably due to RNA editing. These nucleotides were included in the custom-design of the Taqman small RNA assays. An overview of the sequences used for the custom-design Taqman small RNA assays can be found in Table S2.

RT-qPCR

For RNA isolation, the upper 0.5 mL of EV-depleted plasma was used. Prior to RNA isolation, RNA spike-ins UniSp2, UniSp4, and UniSp5 (RNA Spike-in Kit, QIAGEN, cat no. 339390, Venlo, the Netherlands) were added to the samples according to the manufacturer's instruction. Then, RNA was isolated using the miRNeasy Serum/Plasma Kit (QIAGEN, cat no. 217184) according to the manufacturer's instruction.

Reverse transcription of RNA was performed using the high-capacity RNA-to-cDNA reverse transcription kit (Applied Biosystems, Thermo Fisher Scientific, cat no. 4388950) and the tRF specific Taqman RT primer from the custom-design Taqman small RNA assay. cDNA samples were diluted once before performing quantitative PCR. qPCRs were performed on a QuantStudio 5 Real-Time PCR System (Applied Biosystems, Thermo Fisher Scientific) using the custom designed Taqman small RNA assays and Taqman Universal PCR Master Mix, No Amperase UNG (Applied Biosystems, Thermo Fisher Scientific, cat no. 4324018).

Data analysis

Clinical data are represented as mean with standard deviation (SD) or as median with IQR.

Quantitative PCR results were analyzed following the data handling pipeline proposed by de Ronde et al.[44] tRFs were considered undetectable if the average quantification cycle (Cq) from the technical triplets exceeded a threshold of 31, and the value was adjusted to 32. Measures that were labeled as invalid by the data handling pipeline were imputed using multiple imputations. Amplification efficiencies (Figures S4 and S5) for all custom-designed Taqman kits were characterized using serial dilution and incorporated in the relative expression calculations. tRF expression was normalized to an averaged expression of UniSp2, UniSp4, and UniSp5. For statistical analysis, expression data were log-transformed because these were skewed. Statistical differences were assessed using two-sided Student's t tests (ArgTCG$^{53-67}$, LeuCAG$^{53-67}$, LeuCAG$^{54-69}$, SerACT$^{1-15}$, SerGCT$^{1-18}$, SerGCT$^{1-24}$, ThrCGT$^{16-30}$, TyrGTA$^{9-29}$, and TyrGTA$^{1-19}$) or non-parametrical alternative if data remained skewed after log transformation (GlyCCC$^{1-30}$, LeuTAA$^{8-24}$, and ValCAC$^{1-32}$). The hypothesis "groups are equal" was rejected when $p < 0.05$. Analyses were performed using SPSS (v.27.0), and graphs were generated in GraphPad Prism (v.10.2.3).

"Common tRF model" validation

Our pilot study identified a "common tRF model" consisting of isodecoders ThrCGT, TyrGTA, and ValCAC as a predictor for stroke diagnosis. To assess the performance of this model, now consisting of specific fragments derived from these isodecoders, rather than the sum of all fragments, we determined generalized linear model from ThrCGT$^{16-30}$, TyrGTA$^{1-19}$, TyrGTA$^{9-29}$, and ValCAC$^{1-32}$. ROC curves were generated for each separate predictor as well as for the "common tRF model," to assess their predictive ability in distinguishing either IS, ICH, or SM from the other two groups. Analyses were performed using SPSS (v.27.0, IBM).

Regression analyses

LASSO regression analysis was performed to identify a new optimal model to predict stroke diagnosis in our dataset. The optimal model was defined as the model with the lowest cross-validated error. Analysis was performed using the glmnet package in R (v.4.4).

DATA AVAILABILITY

The clinical data presented in this study are available on request from the corresponding author. These data are not publicly available due to privacy regulations.

ACKNOWLEDGMENTS

We thank the students who assisted in this study, particularly Julia van Lent. Additionally, we kindly thank Naomi Wijers for her technical support. We kindly thank Erik W. van Zwet for his guidance/contributions to the statistical methods. Lastly, we thank the HMC Wetenschapsbureau for their support and contribution to this study. The graphical abstract was created using https://Biorender.com. The study was supported by funding from the Leiden Delft Health Initiative and the Netherlands Heart Foundation (CINTICS Program, 2018B031).

AUTHOR CONTRIBUTIONS

T.W., data curation, formal analysis, investigation, methodology, visualization, and writing original article. M.L.v.d.B., data curation, investigation, methodology, and reviewing the article. D.A.L.v.d.H., investigation and reviewing the article. T.T.M.N., data curation, investigation, methodology, and reviewing the article. M.J.H.W., supervision and reviewing the article. I.R.v.d.W., supervision and reviewing the article. P.H.A. Q., supervision and reviewing the article. A.Y.N., conceptualization, funding acquisition, methodology, supervision, and editing and reviewing the article. N.D.K., conceptualization, funding acquisition, methodology, supervision, and editing and reviewing the article.

DECLARATION OF INTERESTS

M.L.v.d.B., T.T.M.N., P.H.A.Q., A.Y.N., and N.D.K. have filed the following patent: Novel biomarkers for use in stroke diagnosis and prognosis. Nossent AY, Quax PH, Kruyt ND, Nguyen TM, van der Bent ML. N2025782. Filed: June 10, 2020.

SUPPLEMENTAL INFORMATION

Supplemental information can be found online at https://doi.org/10.1016/j.omtn.2025.102553.

REFERENCES

1. Saver, J.L. (2006). Time is brain–quantified. Stroke *37*, 263–266. https://doi.org/10.1161/01.STR.0000196957.55928.ab.
2. Emberson, J., Lees, K.R., Lyden, P., Blackwell, L., Albers, G., Bluhmki, E., Brott, T., Cohen, G., Davis, S., Donnan, G., et al. (2014). Effect of treatment delay, age, and stroke severity on the effects of intravenous thrombolysis with alteplase for acute ischaemic stroke: a meta-analysis of individual patient data from randomised trials. Lancet *384*, 1929–1935. https://doi.org/10.1016/S0140-6736(14)60584-5.
3. Berkhemer, O.A., Fransen, P.S.S., Beumer, D., van den Berg, L.A., Lingsma, H.F., Yoo, A.J., Schonewille, W.J., Vos, J.A., Nederkoorn, P.J., Wermer, M.J.H., et al. (2015). A Randomized Trial of Intraarterial Treatment for Acute Ischemic Stroke. N. Engl. J. Med. *372*, 11–20. https://doi.org/10.1056/NEJMoa1411587.
4. Nguyen, T.T.M., van den Wijngaard, I.R., Bosch, J., van Belle, E., van Zwet, E.W., Dofferhoff-Vermeulen, T., Duijndam, D., Koster, G.T., de Schryver, E.L.L.M., Kloos, L.M.H., et al. (2021). Comparison of Prehospital Scales for Predicting Large Anterior Vessel Occlusion in the Ambulance Setting. JAMA Neurol. *78*, 157–164. https://doi.org/10.1001/jamaneurol.2020.4418.
5. H Buck, B., Akhtar, N., Alrohimi, A., Khan, K., and Shuaib, A. (2021). Stroke mimics: incidence, aetiology, clinical features and treatment. Ann. Med. *53*, 420–436. https://doi.org/10.1080/07853890.2021.1890205.
6. Duvekot, M.H.C., Venema, E., Rozeman, A.D., Moudrous, W., Vermeij, F.H., Biekart, M., Lingsma, H.F., Maasland, L., Wijnhoud, A.D., Mulder, L.J.M.M., et al. (2021). Comparison of eight prehospital stroke scales to detect intracranial large-vessel occlusion in suspected stroke (PRESTO): a prospective observational study. Lancet Neurol. *20*, 213–221. https://doi.org/10.1016/S1474-4422(20)30439-7.
7. Logallo, N., Novotny, V., Assmus, J., Kvistad, C.E., Alteheld, L., Rønning, O.M., Thommessen, B., Amthor, K.F., Ihle-Hansen, H., Kurz, M., et al. (2017). Tenecteplase versus alteplase for management of acute ischaemic stroke (NOR-TEST): a phase 3, randomised, open-label, blinded endpoint trial. Lancet Neurol. *16*, 781–788. https://doi.org/10.1016/S1474-4422(17)30253-3.
8. Sohel, M.H. (2016). Extracellular/Circulating MicroRNAs: Release Mechanisms, Functions and Challenges. Achievements in the Life Sciences *10*, 175–186. https://doi.org/10.1016/j.als.2016.11.007.
9. Tosar, J.P., Segovia, M., Castellano, M., Gámbaro, F., Akiyama, Y., Fagúndez, P., Olivera, Á., Costa, B., Possi, T., Hill, M., et al. (2020). Fragmentation of extracellular ribosomes and tRNAs shape the extracellular RNAome. Nucleic Acids Res. *48*, 12874–12888. https://doi.org/10.1093/nar/gkaa674.
10. Tiedt, S., and Dichgans, M. (2018). Role of Non-Coding RNAs in Stroke. Stroke *49*, 3098–3106. https://doi.org/10.1161/STROKEAHA.118.021010.
11. Mens, M.M.J., Heshmatollah, A., Fani, L., Ikram, M.A., Ikram, M.K., and Ghanbari, M. (2021). Circulatory MicroRNAs as Potential Biomarkers for Stroke Risk: The Rotterdam Study. Stroke *52*, 945–953. https://doi.org/10.1161/STROKEAHA.120.031543.
12. Tosar, J.P., and Cayota, A. (2020). Extracellular tRNAs and tRNA-derived fragments. RNA Biol. *17*, 1149–1167. https://doi.org/10.1080/15476286.2020.1729584.
13. Nguyen, T.T.M., van der Bent, M.L., Wermer, M.J.H., van den Wijngaard, I.R., van Zwet, E.W., de Groot, B., Quax, P.H.A., Kruyt, N.D., and Nossent, A.Y. (2020).

Circulating tRNA Fragments as a Novel Biomarker Class to Distinguish Acute Stroke Subtypes. Int. J. Mol. Sci. *22*, 135. https://doi.org/10.3390/ijms22010135.

14. Keam, S.P., and Hutvagner, G. (2015). tRNA-Derived Fragments (tRFs): Emerging New Roles for an Ancient RNA in the Regulation of Gene Expression. Life *5*, 1638–1651. https://doi.org/10.3390/life5041638.
15. Sun, X., Yang, J., Yu, M., Yao, D., Zhou, L., Li, X., Qiu, Q., Lin, W., Lu, B., Chen, E., et al. (2020). Global identification and characterization of tRNA-derived RNA fragment landscapes across human cancers. NAR Cancer *2*, zcaa031. https://doi.org/10.1093/narcan/zcaa031.
16. Fu, X., He, X., Yang, Y., Jiang, S., Wang, S., Peng, X., Tang, G., Zong, T., Li, X., Zhang, Y., et al. (2021). Identification of transfer RNA-derived fragments and their potential roles in aortic dissection. Genomics *113*, 3039–3049. https://doi.org/10.1016/j.ygeno.2021.06.039.
17. Krishna, S., Raghavan, S., DasGupta, R., and Palakodeti, D. (2021). tRNA-derived fragments (tRFs): establishing their turf in post-transcriptional gene regulation. Cell. Mol. Life Sci. *78*, 2607–2619. https://doi.org/10.1007/s00018-020-03720-7.
18. Tiedt, S., Prestel, M., Malik, R., Schieferdecker, N., Duering, M., Kautzky, V., Stoycheva, I., Böck, J., Northoff, B.H., Klein, M., et al. (2017). RNA-Seq Identifies Circulating miR-125a-5p, miR-125b-5p, and miR-143-3p as Potential Biomarkers for Acute Ischemic Stroke. Circ. Res. *121*, 970–980. https://doi.org/10.1161/CIRCRESAHA.117.311572.
19. Cabrelle, C., Giorgi, F.M., and Mercatelli, D. (2024). Quantitative and qualitative detection of tRNAs, tRNA halves and tRFs in human cancer samples: Molecular grounds for biomarker development and clinical perspectives. Gene *898*, 148097. https://doi.org/10.1016/j.gene.2023.148097.
20. Deschamps-Francoeur, G., Simoneau, J., and Scott, M.S. (2020). Handling multi-mapped reads in RNA-seq. Comput. Struct. Biotechnol. J. *18*, 1569–1576. https://doi.org/10.1016/j.csbj.2020.06.014.
21. Johnson, N.R., Yeoh, J.M., Coruh, C., and Axtell, M.J. (2016). Improved Placement of Multi-mapping Small RNAs. G3 (Bethesda) *6*, 2103–2111. https://doi.org/10.1534/g3.116.030452.
22. Nopp, S., Konigsbrugge, O., Schmaldienst, S., Saemann, M., Pabinger, I., Nossent, A. Y., and Ay, C. (2024). Transfer RNAs are Linked to Ischemic Stroke and Major Bleeding in Patients with End-Stage Kidney Disease. Thromb. Haemost. https://doi.org/10.1055/a-2413-2792.
23. Pereira, M., Francisco, S., Varanda, A.S., Santos, M., Santos, M.A.S., and Soares, A.R. (2018). Impact of tRNA Modifications and tRNA-Modifying Enzymes on Proteostasis and Human Disease. Int. J. Mol. Sci. *19*, 3738. https://doi.org/10.3390/ijms19123738.
24. Potapov, V., Fu, X., Dai, N., Corrêa, I.R., Jr., Tanner, N.A., and Ong, J.L. (2018). Base modifications affecting RNA polymerase and reverse transcriptase fidelity. Nucleic Acids Res. *46*, 5753–5763. https://doi.org/10.1093/nar/gky341.
25. van der Kwast, R.V.C.T., Quax, P.H.A., and Nossent, A.Y. (2019). An Emerging Role for isomiRs and the microRNA Epitranscriptome in Neovascularization. Cells *9*, 61. https://doi.org/10.3390/cells9010061.
26. Woudenberg, T., Kruyt, N.D., Quax, P.H.A., and Nossent, A.Y. (2022). Change of Heart: the Epitranscriptome of Small Non-coding RNAs in Heart Failure. Curr. Heart Fail. Rep. *19*, 255–266. https://doi.org/10.1007/s11897-022-00561-2.
27. Jonkhout, N., Tran, J., Smith, M.A., Schonrock, N., Mattick, J.S., and Novoa, E.M. (2017). The RNA modification landscape in human disease. RNA *23*, 1754–1769. https://doi.org/10.1261/rna.063503.117.
28. Fuchs, R.T., Sun, Z., Zhuang, F., and Robb, G.B. (2015). Bias in ligation-based small RNA sequencing library construction is determined by adaptor and RNA structure. PLoS One *10*, e0126049. https://doi.org/10.1371/journal.pone.0126049.
29. Hafner, M., Renwick, N., Brown, M., Mihailović, A., Holoch, D., Lin, C., Pena, J.T.G., Nusbaum, J.D., Morozov, P., Ludwig, J., et al. (2011). RNA-ligase-dependent biases in miRNA representation in deep-sequenced small RNA cDNA libraries. RNA *17*, 1697–1712. https://doi.org/10.1261/rna.2799511.
30. Zhuang, F., Fuchs, R.T., Sun, Z., Zheng, Y., and Robb, G.B. (2012). Structural bias in T4 RNA ligase-mediated 3’-adapter ligation. Nucleic Acids Res. *40*, e54. https://doi.org/10.1093/nar/gkr1263.
31. Scacchetti, A., Shields, E.J., Trigg, N.A., Lee, G.S., Wilusz, J.E., Conine, C.C., and Bonasio, R. (2024). A ligation-independent sequencing method reveals tRNA-derived RNAs with blocked 3’ termini. Mol. Cell *84*, 3843–3859.e8. https://doi.org/10.1016/j.molcel.2024.07.008.
32. Balatti, V., Nigita, G., Veneziano, D., Drusco, A., Stein, G.S., Messier, T.L., Farina, N. H., Lian, J.B., Tomasello, L., Liu, C.G., et al. (2017). tsRNA signatures in cancer. Proc. Natl. Acad. Sci. USA *114*, 8071–8076. https://doi.org/10.1073/pnas.1706908114.
33. Pekarsky, Y., Balatti, V., Palamarchuk, A., Rizzotto, L., Veneziano, D., Nigita, G., Rassenti, L.Z., Pass, H.I., Kipps, T.J., Liu, C.G., and Croce, C.M. (2016). Dysregulation of a family of short noncoding RNAs, tsRNAs, in human cancer. Proc. Natl. Acad. Sci. USA *113*, 5071–5076. https://doi.org/10.1073/pnas.1604266113.
34. Saikia, M., Krokowski, D., Guan, B.J., Ivanov, P., Parisien, M., Hu, G.F., Anderson, P., Pan, T., and Hatzoglou, M. (2012). Genome-wide identification and quantitative analysis of cleaved tRNA fragments induced by cellular stress. J. Biol. Chem. *287*, 42708–42725. https://doi.org/10.1074/jbc.M112.371799.
35. Chen, Y., Tang, Y., Hou, S., Luo, J., Chen, J., Qiu, H., Chen, W., Li, K., He, J., and Li, J. (2023). Differential expression spectrum and targeted gene prediction of tRNA-derived small RNAs in idiopathic pulmonary arterial hypertension. Front. Mol. Biosci. *10*, 1204740. https://doi.org/10.3389/fmolb.2023.1204740.
36. Zhou, H., Kimsey, I.J., Nikolova, E.N., Sathyamoorthy, B., Grazioli, G., McSally, J., Bai, T., Wunderlich, C.H., Kreutz, C., Andricioaei, I., and Al-Hashimi, H.M. (2016). m(1)A and m(1)G disrupt A-RNA structure through the intrinsic instability of Hoogsteen base pairs. Nat. Struct. Mol. Biol. *23*, 803–810. https://doi.org/10.1038/nsmb.3270.
37. Roost, C., Lynch, S.R., Batista, P.J., Qu, K., Chang, H.Y., and Kool, E.T. (2015). Structure and thermodynamics of N6-methyladenosine in RNA: a spring-loaded base modification. J. Am. Chem. Soc. *137*, 2107–2115. https://doi.org/10.1021/ja513080v.
38. Cohen, L., Hartman, M.R., Amardey-Wellington, A., and Walt, D.R. (2017). Digital direct detection of microRNAs using single molecule arrays. Nucleic Acids Res. *45*, e137. https://doi.org/10.1093/nar/gkx542.
39. McArdle, H., Hogg, M.C., Bauer, S., Rosenow, F., Prehn, J.H.M., Adamson, K., Henshall, D.C., and Spain, E. (2020). Quantification of tRNA fragments by electrochemical direct detection in small volume biofluid samples. Sci. Rep. *10*, 7516. https://doi.org/10.1038/s41598-020-64485-4.
40. Ramani, N., Figg, C.A., Anderson, A.J., Winegar, P.H., Oh, E., Ebrahimi, S.B., Samanta, D., and Mirkin, C.A. (2023). Spatially-Encoding Hydrogels With DNA to Control Cell Signaling. Adv. Mater. *35*, e2301086. https://doi.org/10.1002/adma.202301086.
41. Boileau, A., Lino Cardenas, C.L., Lindsay, M.E., and Devaux, Y.; Cardiolinc™ network (2018). Endogenous Heparin Interferes with Quantification of MicroRNAs by RT-qPCR. Clin. Chem. *64*, 863–865. https://doi.org/10.1373/clinchem.2017.284653.
42. Papanagiotou, P., Reith, W., Kastrup, A., and Roth, C. (2014). Current Reperfusion Strategies for Acute Stroke. Interv. Cardiol. Clin. *3*, 145–167. https://doi.org/10.1016/j.iccl.2013.09.008.
43. Williams, L.S., Yilmaz, E.Y., and Lopez-Yunez, A.M. (2000). Retrospective Assessment of Initial Stroke Severity With the NIH Stroke Scale. Stroke *31*, 858–862. https://doi.org/10.1161/01.STR.31.4.858.
44. de Ronde, M.W.J., Ruijter, J.M., Lanfear, D., Bayes-Genis, A., Kok, M.G.M., Creemers, E.E., Pinto, Y.M., and Pinto-Sietsma, S.J. (2017). Practical data handling pipeline improves performance of qPCR-based circulating miRNA measurements. RNA *23*, 811–821. https://doi.org/10.1261/rna.059063.116.

OMTN, Volume *36*

Supplemental information

Validation of tRNA-derived fragments as diagnostic biomarkers in suspected acute stroke; limitations in analysis and quantification methods

Tamar Woudenberg, M. Leontien van der Bent, Daphne A.L. van den Homberg, T. Truc My Nguyen, Marieke J.H. Wermer, Ido R. van den Wijngaard, Paul H.A. Quax, A. Yaël Nossent, and Nyika D. Kruyt

Table S1: Fragments and their contribution to the total reads. Percentages of total reads attributed to the chosen dominant fragment(s), the next most abundant fragment (highlighting the contribution of other prominent tRFs from the same isodecoder) and fragments detected in five or fewer samples (illustrating the proportion of reads derived from sequences with limited occurrence across the patient cohort) are shown. Percentages reflect reads mapped to the same genomic locus/loci as the dominant fragment.

Isodecoder	***Chosen fragment(s):*** *% of Total Reads*	***Next most common fragment:*** *% of Total Reads*	***Rare fragments*** *(<5 samples): % of Total Reads*
ArgTCG	51%	5%	49%
GlyCCC	44%	5%	15%
LeuCAG	12% (8% + 4%)	4%	35%
LeuTAA	17%	9%	23%
SerACT	10%	2%	55%
SerGCT	16% (9% + 7%)	5%	20%
ThrCGT	24%	12%	35%
TyrGTA	7% (4% + 3%)	3%	59%
ValCAC	18%	10%	23%

Table S2: Sequences used for the design of custom Taqman small RNA assays. Between brackets are the nucleotides that were used to extend the original target sequences if the sequence length was beneath 17 nucleotides. Nucleotides in red represent single nucleotide polymorphisms.

**Target**	**Sequence**	**Length**
Arg-TCG$^{53-67}$	5' (GG) GUUCGAAUCCCUCCG 3'	15 (17)
Gly-CCC$^{1-30}$	5' GCAUUGGUGGUUCAGUGGUAGAAUUCUCGC 3'	30
Leu-CAG$^{53-67}$	5' (UG) GGAGGCGUGGGUUCGAA 3'	15 (17)
Leu-CAG$^{54-69}$	5'(G) GAGGCGUGGGUUCGAA 3'	16 (17)
Leu-TAA$^{8-24}$	5' UGGCCGAGUGGUUAAGG 3'	17
Ser-ACT$^{1-15}$	5' (UU) GCCCGGUUAGCUCAG 3'	15 (17)
Ser-GCT$^{1-18}$	5' GACGACGUGGCCGAGUGG 3'	18
Ser-GCT$^{1-24}$	5' GACGACGUGGCCGAGUGGUUAAGG 3'	24
Thr-CGT$^{16-30}$	5' (AG) CGGAAGCGUGCUGGG 3'	15 (17)
Tyr-GTA$^{9-29}$	5' AGCUCAGUUGGUAGAGCGUGG 3'	21
Tyr-GTA$^{1-19}$	5' CUCUCGAUAGCUCAGUUGG 3'	19
Val-CAC$^{1-32}$	5' GUUUCCGUAGUGUAGUGGUUAUCACGUUCGCC 3'	32

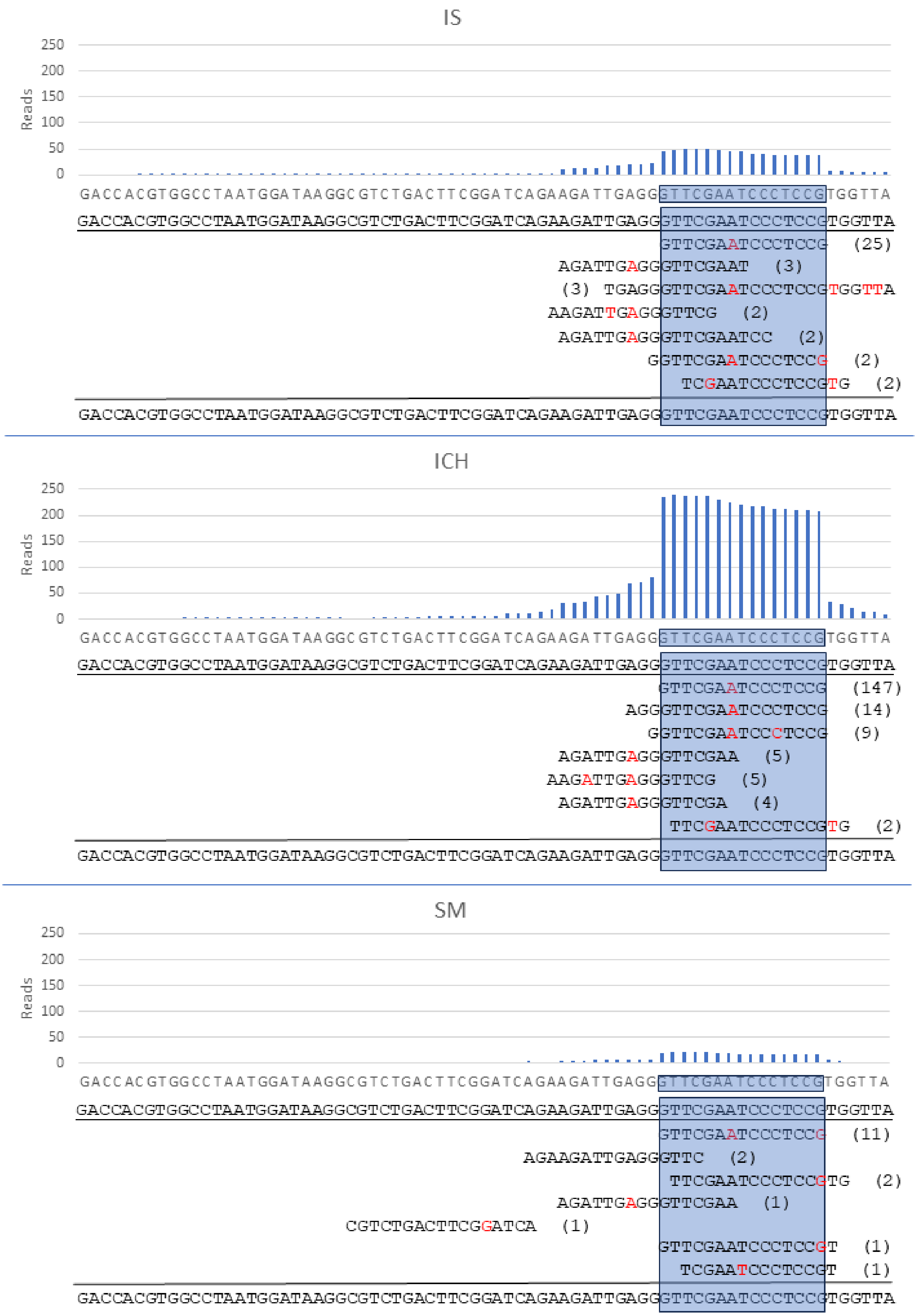

Figure S1: Read density of fragments derived from **ArgTCG** (that were mapped to chr6trna4) in ischemic stroke (IS), intracerebral hemorrhage (ICH) and stroke mimic (SM) patients. The seven most abundant sequences from the RNA sequencing data are displayed with the total amount of reads of that specific sequence between brackets. Indicated in red are the nucleotides that were substituted with a different nucleotide in at least 10% of the reads.

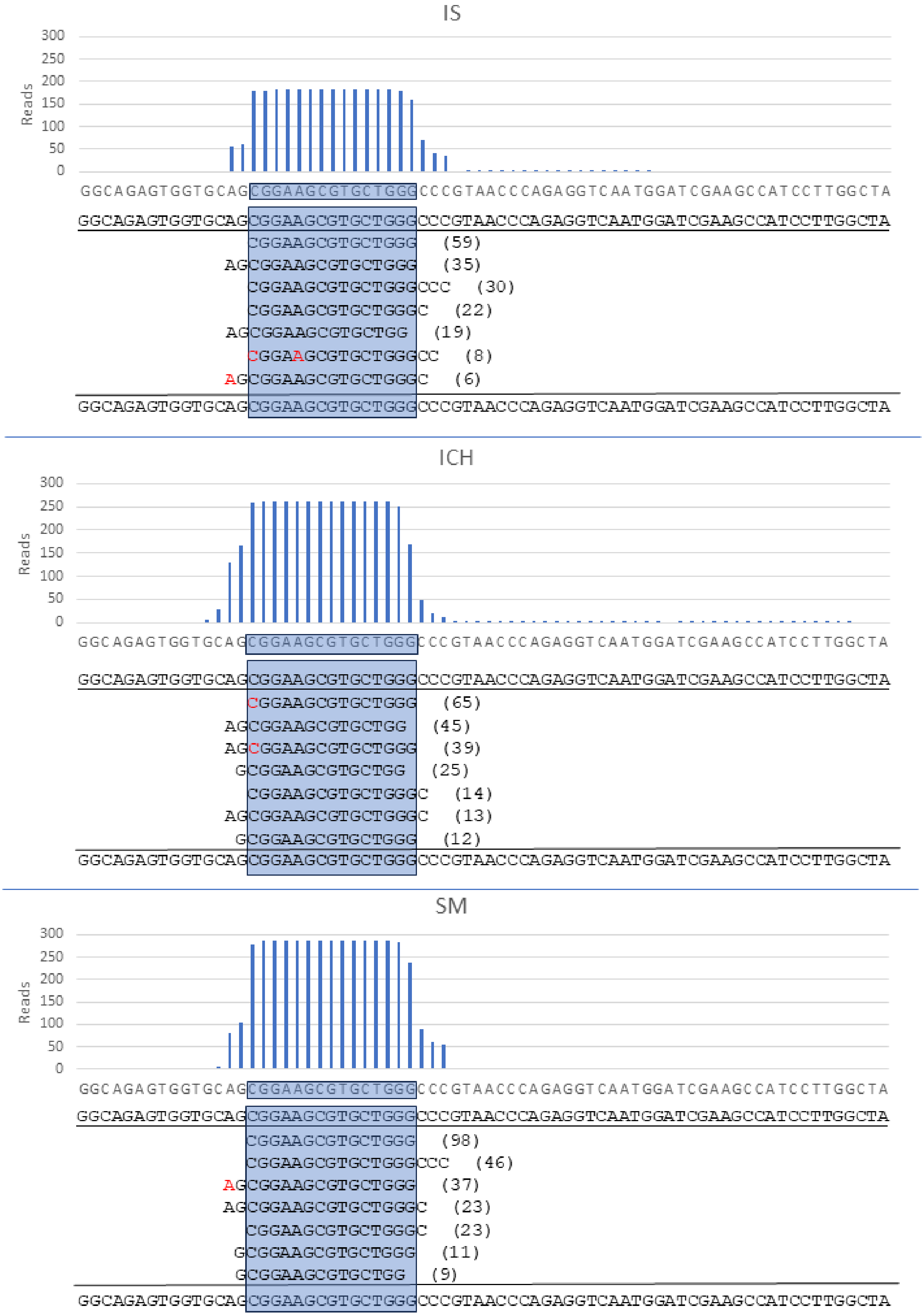

Figure S2: Read density of fragments derived from **ThrCGT** (that were mapped to chr6trna151) in ischemic stroke (IS), intracerebral hemorrhage (ICH) and stroke mimic (SM) patients. The seven most abundant sequences from the RNA sequencing data are displayed with the total amount of reads of that specific sequence between brackets. Indicated in red are the nucleotides that were substituted with a different nucleotide in at least 10% of the reads.

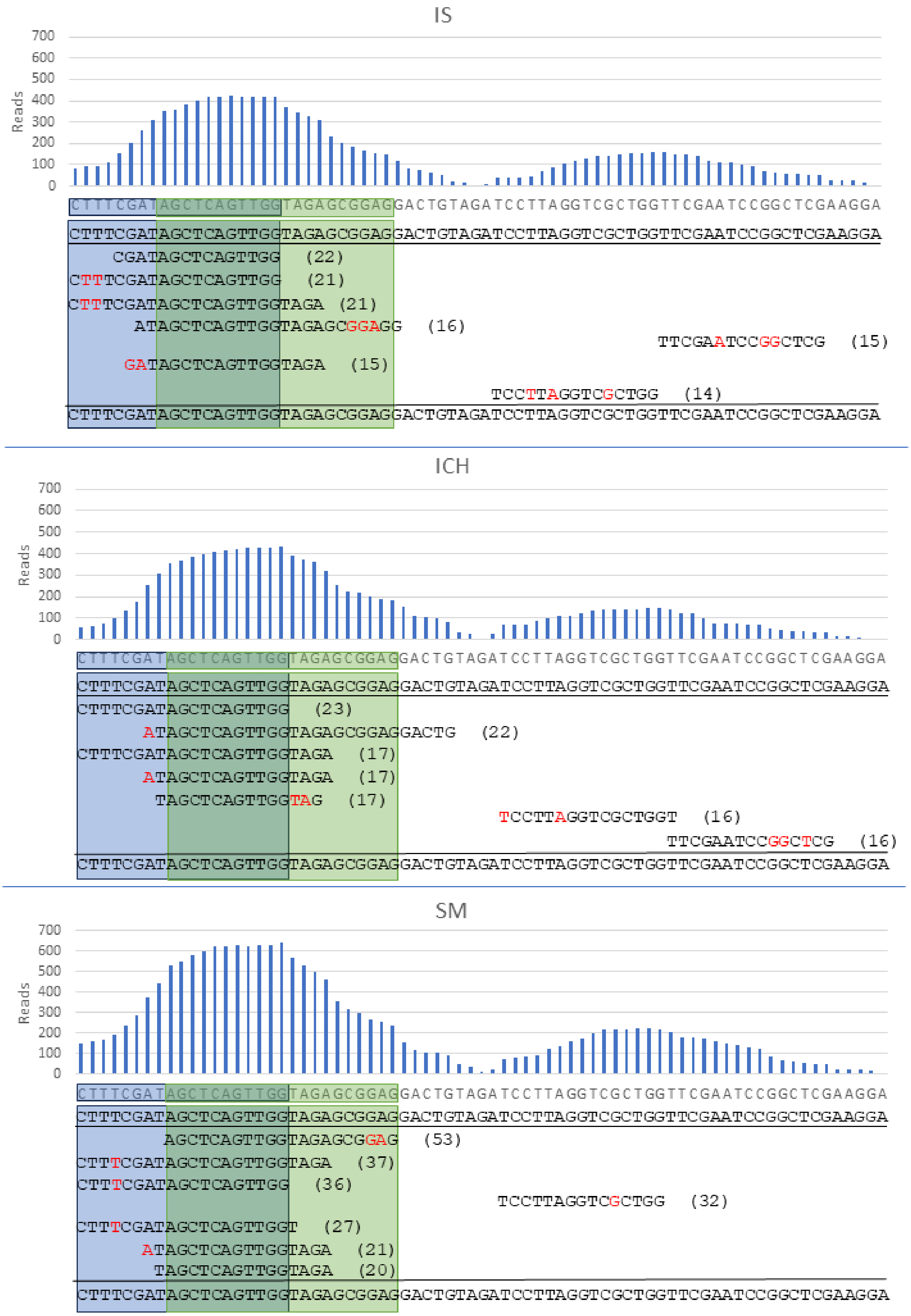

Figure S3: Read density of fragments derived from **TyrGTA** (that were mapped to chr6trna15) in ischemic stroke (IS), intracerebral hemorrhage (ICH) and stroke mimic (SM) patients. The seven most abundant sequences from the RNA sequencing data are displayed with the total amount of reads of that specific sequence between brackets. Indicated in red are the nucleotides that were substituted with a different nucleotide in at least 10% of the reads.

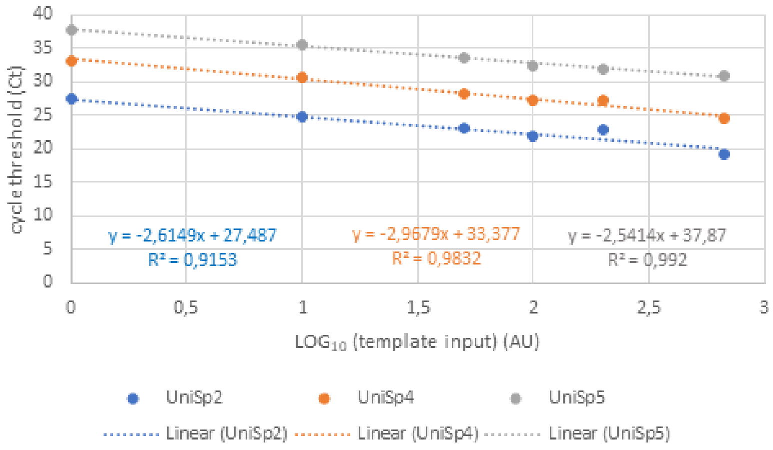

Efficiency:	
UniSp2	2,412275
UniSp4	2,172383
UniSp5	2,474498

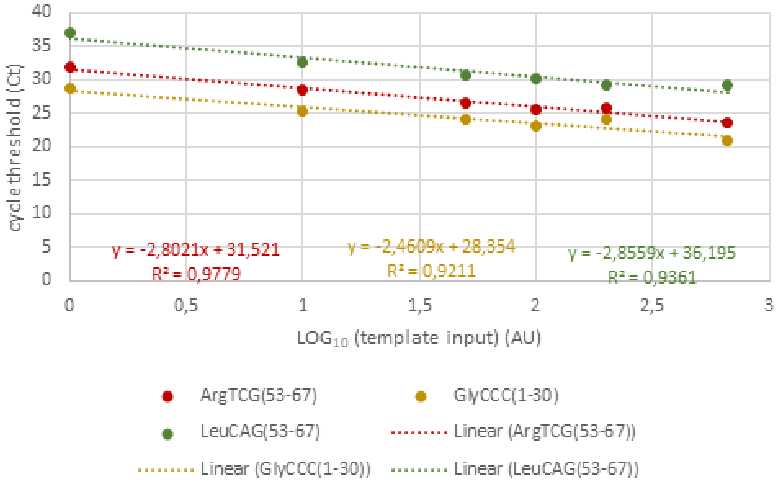

Efficiency:	
ArgTCG$^{53-67}$	2,274442
GlyCCC$^{1-30}$	2,548877
LeuCAG$^{53-67}$	2,239535

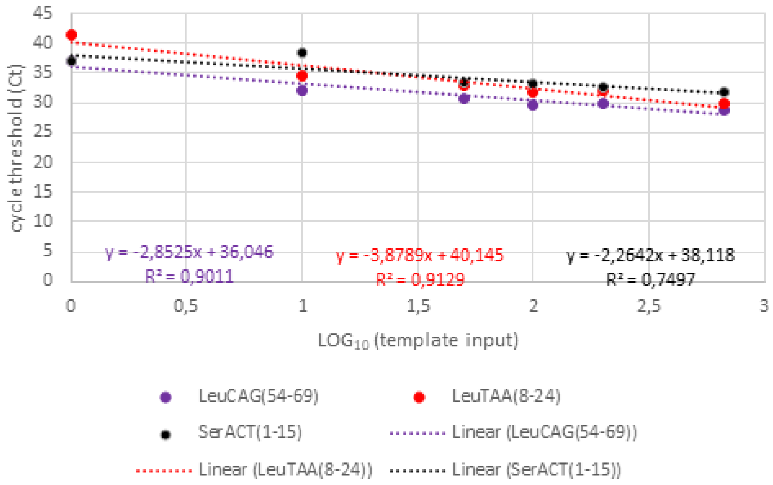

Efficiency:	
LeuCAG$^{54-69}$	2,241679
LeuTAA$^{8-24}$	1,810526
SerACT$^{1-15}$	2,764817

Figure S4: Characterization of qPCR efficiencies of Custom Taqman Small RNA assays for UniSp2, UniSp4, UniSp5, ArgTCG$^{53-67}$, GlyCCC$^{1-30}$, LeuCAG$^{53-67}$, LeuCAG$^{54-69}$, LeuTAA$^{8-24}$, and SerACT$^{1-15}$ by serial dilution of pooled cDNA of patient plasma samples. Cycle threshold was plotted versus the logarithmic function of the relative input. The equation: efficiency = $10^{(-1/slope)}$ was used to calculate the corresponding real-time PCR efficiencies[1].

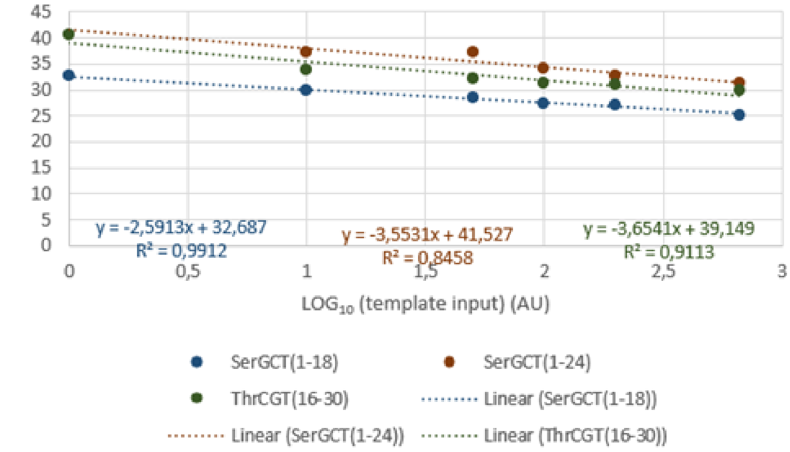

	Efficiency:
SerGCT1	2,431663
SerGCT2	1,911812
ThrCGT	1,877877

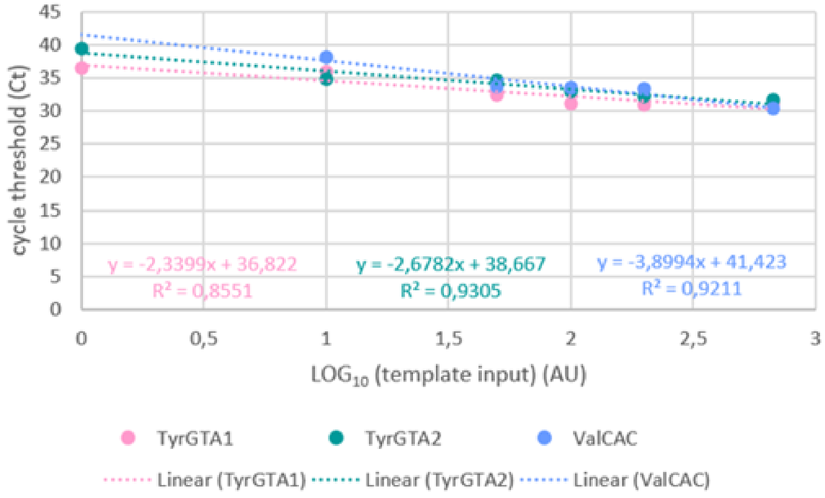

	Efficiency:
TyrGTA1	2,675326
TyrGTA2	2,362552
ValCAC	1,804893

Figure S5: Characterization of qPCR efficiencies of Custom Taqman Small RNA assays for SerGCT$^{1-18}$, SerGCT$^{1-24}$, ThrCGT$^{16-30}$, TyrGTA$^{9-29}$, TyrGTA$^{1-19}$, and ValCAC$^{1-32}$ by serial dilution of pooled cDNA of patient plasma samples. Cycle threshold was plotted versus the logarithmic function of the relative input. The equation: efficiency = $10^{(-1/\text{slope})}$ was used to calculate the corresponding real-time PCR efficiencies[1].

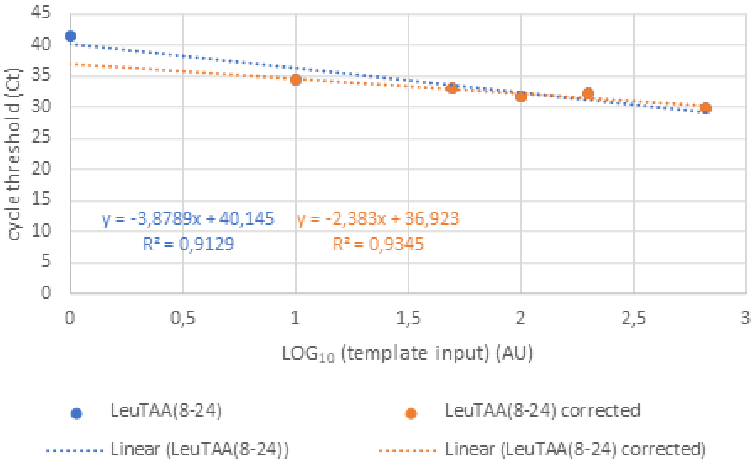

	Efficiency:
$LeuTAA^{8-24}$	1,810526
$LeuTAA^{8-24}$ corrected	2,628083

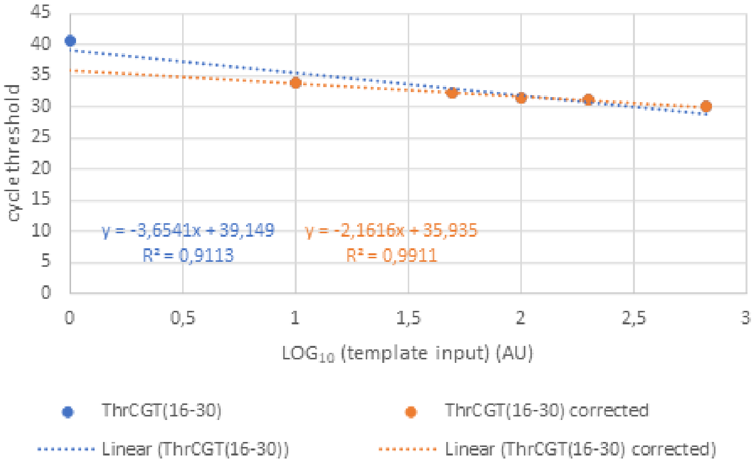

	Efficiency:
$ThrCGT^{16-30}$	1,877877
$ThrCGT^{16-30}$ corrected	2,901485

Figure S6: Characterization of qPCR efficiencies of Custom Taqman Small RNA assays for $LeuTAA^{8-24}$ and $ThrCGT^{16-30}$ by serial dilution of pooled cDNA of patient plasma samples with and without correction for the highest dilution. Cycle threshold was plotted versus the logarithmic function of the relative input. The equation: efficiency = $10^{(-1/slope)}$ was used to calculate the corresponding real-time PCR efficiencies[1].

References:

1. Pfaffl, M.W. (2001). A new mathematical model for relative quantification in real-time PCR. Nucleic Acids Res *29*, 6. 10.1093/nar/29.9.e45.
